# Generating borderline test samples for randomness testers via intelligent optimization and evolutionary algorithms

**DOI:** 10.1038/s41598-026-38020-w

**Published:** 2026-02-04

**Authors:** Peng Gao, Bin Zhang, Ziyuan Wang, Chenglong Li

**Affiliations:** 1 Information Engineering University, Zhengzhou, 450001 China; 2 Institute for Network Sciences and Cyberspace, Tsinghua University, Beijing, 100084 China; 3 School of Computer Science and Technology, Nanjing University of Posts and Telecommunications, Nanjing, 210023 China

**Keywords:** Random number generation, Randomness testing, Genetic algorithm, Large language model, Multi-objective optimization, Engineering, Mathematics and computing, Physics

## Abstract

Ensuring information security heavily relies on high-quality random sequences for encryption keys. Physical entropy sources, despite their use in generating true random sequences, are susceptible to environmental disturbances, necessitating real-time randomness testing to maintain high entropy. However, existing methods for generating test data for real-time randomness testers face significant challenges, including producing sequences that fail to meet specific randomness criteria, constructing borderline sequences with slight non-randomness, and addressing the difficulty of simultaneously violating multiple randomness criteria. This paper introduces a dynamic test data generation framework designed to address these challenges. The framework leverages evolutionary algorithm (EA) to transform the generation of borderline sequences into a multi-constrained optimization problem, where a large language model (LLM) acts as a dynamic parameter adjuster. By analyzing evolutionary trends in population statistics and interacting with evolutionary dynamics through a game-theoretic mechanism, the LLM adaptively adjusts scaling factors and weight coefficients, mitigating the curse of dimensionality in multi-objective optimization and enabling real-time parameter tuning. The experimental results also highlight the high quality of the generated sequences: our approach can generate borderline test data that slightly fail to satisfy the target randomness criteria, yet exhibit statistical properties very similar to those of high-entropy sources under standard test suites. These borderline sequences are fault-detectable and provide challenging, realistic test inputs for classical statistical-test-based real-time randomness testers.

## Introduction

Encrypting critical information is an important method to ensure information security. Encryption operations often require a high-quality random sequence as a key. Therefore, the quality of the random sequence determines the security of the encryption result. The presence of predictable patterns in random sequences can directly compromise the security of the encryption result, as attackers may exploit such regularities to crack and obtain cryptographic keys. To generate high-quality random sequences, modern security systems typically utilize physical entropy sources or deterministic algorithms^1,2^. The former can be called the true random number generators (TRNGs), which use physical phenomena containing random factors (such as circuit thermal noise and quantum fluctuation noise) as an entropy source to generate true random sequences. However, they are susceptible to external environmental influences, which can reduce the randomness. The latter can be called the pseudo-random number generators (PRNGs), which exhibit high stability but can only generate pseudo-random sequences. It means that the most critical challenge in the key generation problem is to ensure the high randomness of the random sequence as the key.

With the rapid development of information technology, people have higher requirements for information security. At the same time, with the rapid development of quantum computing and artificial intelligence techniques, the attack method based on these techniques can crack the key more efficiently, which poses more serious challenges to information encryption. Therefore, the demand for high-entropy true random sequences is increasingly urgent. To generate high-quality random sequences as keys, on the one hand, researchers attempt to discover new physical entropy sources with high randomness and design entropy-enhancement methods to further improve the randomness of sequences generated based on physical entropy sources. On the other hand, people try to design new deterministic algorithms for generating high-quality pseudo-random numbers, which often utilize the results of physical entropy sources as seeds. It means that the quality of the physical entropy source is usually the determining factor for the quality of true random or pseudo-random sequences. Unfortunately, physical entropy sources are susceptible to environmental disturbances, leading to a decrease in output quality. This phenomenon requires us to perform real-time randomness testing on the random sequences generated from physical entropy sources to ensure that they are in a high-entropy state when using these sources to generate random sequences^3^.

The existing methods for randomness testing primarily fall into two categories: statistical testing methods and prediction-based entropy estimation methods. Statistical testing could verify whether the random sequence meets statistical randomness standards by utilizing mathematical tools to quantitatively analyze random sequences in terms of distribution uniformity, independence, and unpredictability^4^ . NIST firstly proposed a standard set of statistical testing methods^5^, including 0-1 frequency testing, ordered pair testing, poker testing, autocorrelation testing, run testing, etc. NIST also released the statistical testing suite SP800-22^6^, which has become an important benchmark for randomness testing. Classic statistical test suites such as Diehard^7^ and TestU01^8^ are also based on the NIST standard. These statistical testing methods can not only be used to evaluate the randomness of random sequences, but also to guide the improvement of the quality of random sequences. The prediction-based entropy estimation method evaluates the quality of a random sequence and the quality of the entropy source based on whether the random sequence can be predicted and the degree to which it can be accurately predicted^9^. Kelsey et al. first proposed four prediction-based entropy estimators (predictive factors) in 2015. Zhu et al., Kumar et al.^10^, and Fan et al. have attempted to use classic machine learning methods to learn the features of pseudo-random sequences generated by pseudo-random number generators. Yang et al., Zhu et al., and Truong et al. used deep neural networks to predict pseudo-random sequences, thereby evaluating random number generators.

As mentioned in the second paragraph of the previous text, real-time randomness testing is required^11,12^. A real-time randomness tester should detect issues with the incorrect state of the entropy source and the decreasing quality of the random sequence. Such a real-time randomness tester has a high-quality requirement. Because once the real-time randomness checker fails to detect issues with the incorrect state of the entropy source and the decreasing quality of the random sequence, it can easily lead to security incidents such as key guessing and ciphertext cracking. To guarantee the quality of the real-time randomness tester, it is necessary to conduct sufficient testing. High-quality test data is required in the testing to attack the real-time randomness tester and verify whether it can detect issues with the incorrect state of the entropy source and the decreasing quality of the random sequence. Unfortunately, to the best of our knowledge, the techniques for generating test data for real-time randomness testers are still lacking.

The generation of test data for testing a real-time randomness tester presents three key challenges. (1) Generation of specific types of test data. The test data that can successfully attack the real-time randomness tester should be a pseudo-random sequence that fails to meet randomness requirements. Several criteria are usually used to estimate whether a sequence satisfies randomness, including the uniformity and independence of the 0-1 distribution, the ordered pair distribution, and the runs distribution. A sequence fails to satisfy randomness if and only if at least one criterion is not satisfied, which may include different situations, such as the sequence not satisfying only one criterion, not satisfying two criteria, or even not satisfying any of the criteria. (2) Generation of borderline test data. For real-time randomness testers, detecting sequences that fail to satisfy randomness significantly (such as all 0 sequences or all 1 sequences) is not difficult at all. To more accurately evaluate the quality of real-time randomness testers, the test data we use should be sequences that fail to satisfy randomness slightly, rather than sequences that fail to satisfy randomness significantly. Only when a real-time randomness tester can detect borderline sequences that fail to satisfy randomness slightly can the tester be considered high-quality. (3) Our theoretical analysis (see section “Methods”) indicates that constructing a borderline sequence that fails to satisfy multiple criteria simultaneously is highly challenging. The difficulty increases with the number of unsatisfactory criteria.

To address the above challenges, this paper focuses on the problem of generating high-quality test data for testing real-time randomness testers. We propose the Algorithm Parameter Adjustment Mechanism Based on Intelligent Game with Large Language Models (APAM-IGLLM), a dynamic test data generation framework that can generate borderline samples that fail to satisfy one or more specific criteria by utilizing evolutionary algorithms (EAs) and large language models (LLMs). The APAM-IGLLM utilizes genetic algorithms to generate test data by transforming the problem of generating borderline sequences that fail to satisfy the specified criteria into an optimisation problem that approximates the borderline state under these criteria.

In the key parameter tuning component of the genetic algorithm, a large language model is introduced as a dynamic parameter adjuster. Leveraging its advantages in contextual understanding, the LLM analyses the evolutionary trends of population statistical indicators and their relationships with parameters through iterative updates of historical population evolution information. Through a game-theoretic mechanism with the population evolution trends, it intelligently provides real-time adjustment strategies for scaling factors and weight coefficients, thereby addressing the curse of dimensionality in multi-objective optimisation and the challenge of dynamic parameter adjustment during the solution process. Unlike conventional metaheuristic optimisation methods such as MSDCS^13^, which rely on hand-crafted schedules and local statistics for continuous single-objective optimisation, our framework operates directly in the discrete space of binary sequences and dynamically reshapes a multi-metric fitness landscape under five tightly coupled randomness tests. Moreover, in contrast to model-driven signal decomposition approaches such as VFW-VMD^14^ and compression-oriented block selection frameworks like BLOTTER^15^, which optimise continuous variational formulations or submodular storage objectives with fixed algorithmic parameters, our framework does not optimise existing signals or compression configurations. Instead, it focuses on the optimisation-driven synthesis of new bit sequences with prescribed borderline statistical behaviours.

Furthermore, while recent security-oriented optimisation and learning frameworks–such as hybrid game and stochastic robust optimisation for multi-integrated energy microgrids^16^, multiobjective structural optimisation of pressure-maintaining ball valves^17^, and deep reinforcement learning based cyber deception deployment^18^–focus on optimising control strategies, structural parameters, or defence policies for given systems, our framework uses optimisation in a different way: to generate adversarial borderline randomness-test samples. A large language model is employed as a high-level, history-aware controller that observes the trajectory of multiple statistical tests and adaptively reconfigures their weights and scaling factors in the fitness function, thereby enhancing the problem-solving ability of evolutionary algorithms in this discrete, highly constrained setting.

Recent studies further demonstrate the effectiveness of optimisation and adaptive mechanisms in diverse engineering and security-related domains. For instance, Guo et al.^19^ embed Gaussian-process-based Bayesian optimisation into a planetary rover control loop to adapt the parameters of a sweeping–spinning gait and maximise forward distance on deformable terrain in real time. Li et al.^20^ introduce an iterative mini-minimum spanning tree (mini-MST) framework for unsupervised outlier detection in medical datasets, using threshold-scaled distances and adaptive exit conditions to handle clusters with varying densities. In manufacturing, Zhang et al.^21^ combine machine learning with a high-throughput NSGA-II to optimise femtosecond laser trepan drilling with respect to taper and processing time. At the model and representation level, Yan et al.^22^ propose a weighted transferable random forest for generalisable NILM via incremental structure expansion and reduction on edge devices, while Chen et al. ^23^ design a recoding scheme for hybrid stochastic numbers that alleviates bit-width accumulation and preserves fault tolerance in stochastic computing circuits.

These works highlight the power of optimisation, online adaptation, and robust stochastic representations. However, they focus on improving the performance or robustness of a given control system, classifier, or numerical representation. In contrast, our goal is fundamentally different: we use optimisation and intelligent parameter adaptation to *generate* statistically controlled borderline random sequences that slightly violate multiple randomness tests, thereby providing adversarial test data for evaluating the robustness of real-time randomness testers.

The experimental results also demonstrate the high quality of the generated sequences: (1) statistical testing shows that the generated sequences fall within borderline intervals (1.0–1.3 times the threshold) under all criteria, indicating that the proposed technique can generate test data that slightly violate the target randomness requirements while remaining close to the acceptance boundary; (2) entropy evaluation shows that the min-entropy values of the generated sequences are very close to those of commonly used commercial entropy sources, indicating that, from the viewpoint of statistical-test-based real-time randomness detectors, the generated borderline data can effectively mimic high-quality random inputs and serve as fault-detectable, stress-testing cases.

Our research provides high-precision testing tools for scenarios such as statistical-test-based commercial security device evaluation and hardware entropy-source monitoring, thereby expanding the application scope of such techniques.

The contributions of this paper mainly include:

- The first attempt to conduct testing for the real-time randomness testers that are used to evaluate the quality of physical entropy sources and the randomness of pseudo-random sequences.
- Define five borderline criteria to evaluate the degree to which pseudo-random sequences that fail to satisfy randomness are difficult to be determined as non-random sequences.
- Propose a test data generation technique for generating borderline samples under specified criteria. An evolutionary algorithm is employed to conduct solution space search in multi-constrained scenarios, while an LLM is utilized to adjust scaling factors and weight coefficients adaptively. Unlike MSDCS-like metaheuristics that use fixed heuristic schedules for parameter adaptation in continuous single-objective optimisation, and unlike VFW-VMD and BLOTTER, which optimise variational decomposition models or submodular compression objectives without external controllers, our parameter adjustment mechanism is history-aware and multi-metric: the LLM observes the trajectory of five strongly correlated statistical tests and dynamically reconfigures their weights and scaling factors, significantly enhancing the problem-solving ability of evolutionary algorithms in this discrete, highly constrained setting. This LLM-guided parameter regulation goes beyond conventional EA-based or constraint-handling methods with fixed or heuristic penalty schedules: by exploiting multi-test statistics and short-term history, it significantly improves both the proportion of borderline qualified sequences and the convergence speed, as demonstrated in our experiments.
- Conduct quantitative evaluation of the quality of the test data generated by our proposed technique through experiments that include statistical testing and validation of randomness testers.

## Literature survey

### Statistical testing of static entropy sources

Mathematically, it is impossible to directly prove that a numerical sequence generated by an entropy source is truly random. Instead, statistical testing serves as the primary means to assess whether the entropy source exhibits specific vulnerabilities. Typically, multiple statistical test methods are employed to evaluate the output sequences of the entropy source. Each statistical test determines whether the sequence possesses a specific attribute characteristic of a truly random sequence. The conclusions drawn from individual tests are inherently non-deterministic but carry substantial probabilistic confidence. During testing, if a sequence fails to pass a particular statistical test, it is classified as non-random. Conversely, if a sequence passes all applied statistical tests, it is deemed random, and the hypothesis that “the sequence is random” is accepted (or more precisely, “not rejected”).

Statistical testing of Random Number Generators (RNGs) traces its early roots to the implementation of the Diehard CD-ROM Tests in the 1990s^24^. Since then, two primary research directions have emerged. The first involves the development of alternative test suites, such as the NIST Statistical Test Suite^6^ and the TestU01 Suite^8^.The second line of inquiry focuses on empirical testing of True Random Number Generators (TRNGs). For instance, Hamburg et al. analyzed the statistical properties of the entropy source in Intel’s Ivy Bridge TRNG^25^, while subsequent works explored the implementation, evaluation, and statistical testing of diverse TRNGs–including Intel’s random number generator architecture^26^, Field-Programmable Gate Array (FPGA)-based Physical Random Number Generators (PRNGs)^27^, a high-speed physical generator utilizing optical amplifiers to produce spectrally incoherent light for generating 12.5 GB/s high-quality random numbers^28^, and a random number generation method for in-body Internet of Things (IoT) devices that leverages time-signal variations from Inertial Measurement Units (IMUs) during user walking^29^. These efforts have further extended to Quantum Random Number Generators (QRNGs), as demonstrated in studies such as^30–33^, where extensive statistical testing was conducted. Additionally, general-purpose RNG statistical testing frameworks have been proposed, such as the work in^34^. Collectively, these contributions have yielded robust methodologies for static entropy source statistical testing.Table 1 describes the types of statistical tests involved in this study and their corresponding mathematical expressions, and presents the boundary state through normalization processing.

**Table 1:** Mathematical descriptions and normalization processing methods of randomness statistical test items.

No.	Test Item	Statistic	Normalization	Description
1	Frequency Test	$$\begin{aligned} X_1&= \frac{(n_0 - n_1)^2}{n} \end{aligned}$$	$f_1(X_1) = \frac{X_1}{\chi _{1,\alpha }^2}$	$n_0$: number of 0s; $n_1$: number of 1s; $\chi _{1,\alpha }^2$: chi-squared borderline value at significance level $\alpha$ with 1 degree of freedom.
2	Ordered Pair Test	$$\begin{aligned} X_2 &= \frac{4}{n-1}\sum _{i=1}^4 n_i^2 \\ & - \frac{2}{n}(n_0^2 + n_1^2) + 1 \end{aligned}$$	$f_2(X_2) = \frac{X_2}{\chi _{2,\alpha }^2}$	$n_i$: counts of four pairs (00, 01, 10, 11).
3	Poker Test	$$\begin{aligned} X_3&= \frac{2^m}{k}\left( \sum _{i=0}^{2^m-1} n_i^2\right) - k \end{aligned}$$	$f_3(X_3) = \frac{X_3}{\chi _{4,\alpha }^2}$	*m*: group length; $n_i$: counts of the *i*-th group.
4	Auto-relation Test	$$\begin{aligned} X_4&= \frac{2\left( A(d) - \frac{n-d}{2}\right) }{\sqrt{n-d}} \end{aligned}$$	$$\begin{aligned} f_4(X_4) & = {\text {count}} \\ & \left( \chi _{4,\alpha }^2< |X_4| < (1+\gamma )\chi _{4,\alpha }^2\right) \end{aligned}$$	*A*(*d*): correlation statistic at shift *d*.
5	Run Test	$$\begin{aligned} X_5 &= \sum _{i=1}^k \frac{(B_i - e_i)^2}{e_i} \\ & + \sum _{i=1}^k \frac{(G_i - e_i)^2}{e_i} \end{aligned}$$	$f_5(X_5) = \frac{X_5}{\chi _{5,\alpha }^2}$	$e_i$: expected values of 0-runs ($B_i$) and 1-runs ($G_i$).

### Test requirements for embedded randomness testing modules

Industries with stringent information security requirements, such as finance, impose high standards on security devices. Server-type security devices often integrate entropy sources to stably generate random sequences, which undergo a series of entropy enhancement transformations to produce reliable random numbers for cryptographic key generation. However, factors such as physical environmental variations, design flaws, or anomalous operational conditions may lead to degradation of entropy in the entropy source, resulting in poor randomness of the generated random sequences. Directly using such defective random sequences as raw inputs for further transformations would render their reliability insufficient to meet cryptographic security requirements, thereby posing significant security risks.

Consequently, standards such as FIPS^11^ and GM/T^12^ impose specific requirements on random number generators (RNGs) in such security devices, including mandatory power-on testing and conditional testing. These standards mandate the embedding of testing modules within devices to inspect sampled random sequences, aiming to confirm the current state of the entropy source. If entropy source defects are detected, the device should issue alerts or terminate security services as required to mitigate further information security risks. During the testing of such inspection functionalities, validation of potential defect patterns and construction of abnormal test cases are necessary. However, due to the difficulty in generating samples with specific defect patterns, the sufficiency of related testing remains challenging to achieve.

### Typical application scenarios

Cryptographic device certification (e.g., standards such as FIPS 140-3 and GM/T) mandates rigorous testing of the randomness evaluation modules in such devices. Existing methods fail to effectively validate the consistency of test implementation and threshold settings, nor do they adequately conduct in-depth validation of test robustness and consistency under near-failure conditions^5,35^. Hardware entropy sources (e.g., sensors and physical noise sources) may compromise their entropy quality due to degradation or malfunctions. Testing requires constructing test sequences with specific statistical properties to effectively simulate various hardware failure modes^36^. Blockchain technology widely employs Verifiable Random Functions (VRFs) to achieve distributed consensus^37^. However, there remains a lack of test cases for evaluating the robustness of VRF implementations under statistical boundary conditions. Intrusion Detection Systems (IDSs) based on randomness analysis to detect anomalous behaviors lack borderline sequences for calibrating system detection thresholds and analyzing the trade-off between false positives and false negatives^38^.

### Comparison with related optimisation-driven, online, and security-oriented methods

In addition to classical statistical testing frameworks for random number generators, recent research has explored optimisation-driven techniques and adaptive mechanisms in both general metaheuristic algorithms and security-related application domains. In this subsection, we discuss several representative works and contrast them with our APAM-IGLLM framework from the perspectives of problem setting, optimisation objective, and the role of parameter adaptation.

#### Metaheuristic optimisation and adaptive modelling

Cai and Zhang^13^ propose MSDCS, a multi-strategy variant of the Differentiated Creative Search (DCS) algorithm. MSDCS integrates three key strategies: (i) a collaborative development mechanism that incorporates an estimation distribution model to guide exploration; (ii) a population evaluation strategy that balances fitness and distance to coordinate exploration and exploitation; and (iii) a linear population size reduction scheme that gradually reduces the population size over time. These strategies significantly improve the performance of DCS on CEC benchmark functions and engineering optimisation problems. However, MSDCS is designed for continuous single-objective (or conventionally constrained) optimisation, where the algorithm outputs a best solution vector that minimises a scalar objective. Its adaptive behaviours are governed by hand-crafted schedules and local statistics, such as a deterministic evolution of the score-balancing factor and a predefined linear population size schedule. There is no external learning agent or history-aware decision-maker.

In contrast, our APAM-IGLLM framework addresses a discrete, high-dimensional, multi-metric optimisation problem, where each individual encodes a binary sequence and the goal is to enforce five tightly coupled randomness-test statistics to fall into predefined borderline intervals. Instead of only improving the convergence speed of a single objective, we must dynamically balance conflicting statistical metrics that exhibit strong correlations (as shown by our Monte Carlo analysis in section “Methods”). To this end, we introduce a large language model as a high-level controller that observes the trajectory of multiple test statistics over generations, maintains a short-term memory of past parameter settings and rewards, and adaptively adjusts the weights and scaling factors in the fitness function. This turns parameter tuning into a history-aware decision process tailored to the structure of the statistical testing problem, which goes beyond the static heuristic adaptation considered in MSDCS.

Li and Wang^14^ present the Variable Filtered-Waveform Variational Mode Decomposition (VFW-VMD) method for rolling bearing fault feature extraction. Starting from the classical variational mode decomposition framework, they replace the standard second-order derivative regularisation with a fractional-order derivative, yielding a family of Wiener filters with variable waveforms parameterised by the decomposition order. An iterative algorithm based on the alternating direction method of multipliers is then derived to update the modes and their centre frequencies. The main contribution is an improved variational signal decomposition model that better adapts to broadband and chirp signals, thereby reducing mode mixing and over-smoothing.

Although both VFW-VMD and our work use optimisation formulations, their roles and targets are very different. VFW-VMD solves a continuous variational optimisation problem to decompose a given signal; the decomposition parameters (such as the decomposition order and penalty factor) are chosen based on prior knowledge and empirical tuning. The method does not incorporate an intelligent controller to adapt these parameters online. By contrast, our framework does not aim to improve the decomposition quality of a fixed signal. Instead, we treat the bit sequences themselves as optimisation variables and use evolutionary search combined with an LLM-driven parameter adjustment mechanism to generate new sequences that satisfy multi-metric borderline randomness-test constraints. Thus, we focus on optimisation-driven adversarial data generation under multiple statistical constraints rather than on designing a richer variational model for signal decomposition.

Qi et al.^15^ propose BLOTTER, a block-based lossless compression method for highway structural health monitoring (SHM) data. The authors exploit two domain-specific properties of SHM data–slight variation and non-uniform value frequency–and formulate a data block selection problem under file-size constraints. The objective is to minimise the total size of compressed files when first-order differencing and Huffman coding are applied. They prove that this objective is an increasing submodular function and develop a greedy approximation algorithm with a provable approximation ratio. In BLOTTER, the decision variables are block-to-file assignments for pre-existing SHM time series, and the optimisation goal is to reduce storage cost.

BLOTTER shares with our work the use of an optimisation perspective, but it addresses a completely different task. The compression modules and their parameters are static, and the greedy algorithm follows a fixed selection rule; no intelligent or history‑aware parameter adaptation is involved. In our case, optimisation is not used to compress given data but to generate new bit sequences with prescribed borderline statistical behaviours. The evolutionary algorithm operates directly in the space of binary sequences, and the fitness function is dynamically shaped by an LLM‑based parameter adjustment mechanism that uses short‑term memory and reward signals to coordinate five strongly coupled randomness‑test metrics. This type of optimisation‑driven adversarial data generation with intelligent parameter adaptation is not covered by BLOTTER or the above methods. Related enhanced metaheuristic frameworks proposed for other application domains^39–41^ also focus on refining optimisers or models to improve task performance, whereas our approach uses a history‑aware LLM controller specifically to steer evolutionary search towards borderline and defect test sequences for real‑time randomness testers under tightly coupled statistical constraints.

#### Security-oriented optimisation and learning frameworks

Beyond general metaheuristic optimisation, several recent works have applied optimisation and learning techniques in security-related or optimisation-enabled domains.

Gao et al.^16^ propose a bi-level hybrid game framework for stochastic robust optimisation in multi-integrated energy microgrids. Integrated energy microgrids are modelled as leaders and multi-energy loads as followers, while profit allocation among microgrids is governed by a Nash game. Two-stage stochastic robust optimisation is employed to handle uncertainties in renewable generation, multi-energy loads, and electricity prices. Initial scenarios for uncertain variables are generated by a spectrally normalised conditional generative adversarial network, and the resulting optimisation problem is solved using a combination of the alternating direction method of multipliers and a column-and-constraint generation algorithm. This work focuses on scheduling and profit allocation in integrated energy systems; the decision variables are continuous power and pricing quantities, and generative adversarial networks are used to produce uncertainty scenarios for a stochastic robust optimisation model.

In contrast, our APAM-IGLLM framework does not optimise the operation of energy systems or economic objectives. Each individual in our approach encodes a binary sequence, and the optimisation objective is to generate sequences whose empirical behaviour on five randomness tests lies within pre-defined borderline intervals. The sequences themselves are adversarial test samples for real-time randomness testers. Furthermore, while Gao et al. use generative adversarial networks to model uncertainty within a fixed optimisation pipeline, we employ a large language model as a high-level controller that dynamically adjusts metric weights and scaling factors in the fitness function based on the evolution of multiple statistical metrics. In other words, we use optimisation both to generate adversarial data and to adapt the optimisation process itself via an external intelligent controller.

Wen et al.^17^ present a multiobjective optimisation method for the structural design of pressure-maintaining ball valves in deep pressure-preserved coring tools. They establish theoretical and numerical models for valve pressure resistance, identify key structural parameters through sensitivity analysis, construct regression models using response surface methodology and central composite design, and apply NSGA-II to obtain Pareto-optimal structural dimensions with respect to maximum stress and effective sealing width. This is a typical continuous engineering design optimisation problem, where the decision variables are geometric dimensions and the objectives are physical performance indicators. NSGA-II is used with standard, fixed parameter settings to improve structural design.

By contrast, our framework does not optimise mechanical structures or continuous design parameters. We optimise directly in the discrete space of bit sequences to generate new data samples that intentionally exhibit borderline deviations on multiple randomness tests. Our optimisation problem is governed by five strongly coupled statistical constraints, and the main challenge is to balance these metrics so that all of them fall into narrow borderline intervals simultaneously. To address this, we integrate a large language model as a history-aware controller that observes the trajectory of multiple test statistics and adapts the parameters of the evolutionary algorithm, rather than using a fixed metaheuristic configuration.

He et al.^18^ propose a cyber deception resource deployment method based on a time-delay differential game and deep reinforcement learning. They model the evolution of node security states in a scale-free network using time-delay differential equations, formulate a cyber deception time-delay differential game between attackers and defenders, and transform it into a Markov game that is solved by a deep reinforcement learning algorithm based on proximal policy optimisation. The result is an optimal deployment strategy for honeypots of different interaction levels in complex network environments. This work addresses security-oriented decision optimisation: the optimisation variables are deception deployment strategies, and the objective is to maximise defence effectiveness over time.

In our setting, we do not learn defence or attack policies in a live network; instead, we use optimisation to synthesise borderline random sequences that stress and evaluate real-time randomness testers embedded in security-critical devices. Our search space is the discrete domain of bit sequences, and the optimisation objective is to shape their statistical behaviour under multiple randomness tests. Furthermore, while He et al. use deep reinforcement learning to train a policy network under a fixed game model and reward structure, our large language model acts as a meta-level controller that adapts the parameter configuration of the evolutionary search itself in a history-aware manner, coordinating multiple statistical metrics during the search process.

Overall, these optimisation-driven and security-oriented frameworks demonstrate the usefulness of optimisation and intelligent algorithms in metaheuristic search, signal decomposition, compression, energy scheduling, structural design, and cyber deception. Our work is complementary: we focus on optimisation-driven generation of borderline randomness-test samples under multiple strongly coupled statistical constraints in a discrete sequence space, and we employ a large language model as a high-level controller for dynamic parameter adjustment within an evolutionary search framework.While MSDCS, VFW-VMD and BLOTTER are representative of optimisation-driven modelling or data selection with mostly rule-based or locally adaptive parameter schedules, our APAM-IGLLM framework represents a different form of optimisation-driven *data generation* combined with *intelligent parameter adaptation*: the bit sequences themselves are optimisation variables, and a large language model acts as a history-aware controller to adjust the optimisation dynamics under multiple randomness-test constraints. Compared with conventional EA-based or constraint-based sequence generation methods, which typically employ fixed or hand-tuned penalty terms and treat each test independently, APAM-IGLLM introduces an explicit large-language-model controller that reasons over joint multi-test statistics and short-term history to produce coordinated parameter updates. This design yields a fundamentally different optimisation loop and constitutes the core methodological novelty of our work.

#### Online optimisation, outlier detection, and robust stochastic methods

Beyond general metaheuristic optimisation and security-oriented frameworks, several recent works address online optimisation, fault detection, and robustness from perspectives that are complementary to ours.

Guo et al.^19^ propose an online escape-entrapment strategy for planetary rovers based on Bayesian optimisation. They treat the rover’s forward distance under a sweeping–spinning gait as a black-box function of six continuous control parameters and use a Gaussian-process surrogate with an upper confidence bound acquisition function to adapt gait parameters online on loose granular terrain. Their approach exemplifies black-box optimisation embedded in a physical control loop under stringent resource and safety constraints. In our setting, we also optimise a black-box objective, namely the multi-metric fitness of candidate bit sequences under several randomness tests. However, we operate in a high-dimensional *discrete* space of binary sequences and must satisfy multiple tightly coupled statistical boundary constraints, rather than maximising a single continuous performance metric for a physical system.

Li et al.^20^ introduce an iterative adaptive mini-minimum spanning tree (MMOD) algorithm for unsupervised outlier detection in medical data. By constructing a sequence of mini-MSTs using a threshold-scaled Euclidean distance and adaptive exit conditions, MMOD can identify outliers without prior knowledge of the outlier proportion and handles clusters with varying densities. Conceptually, MMOD and related methods aim to *detect* anomalous points in an existing dataset by exploiting geometric and density structure. In contrast, our APAM-IGLLM framework focuses on *actively generating* anomalous-but-high-entropy sequences: we synthesise borderline random sequences that lie near the decision boundaries of multiple randomness tests, which can be seen as intentionally crafted “statistical outliers” for stress-testing real-time randomness testers.

In the context of laser processing, Zhang et al.^21^ combine machine learning with a high-throughput multi-objective genetic algorithm (NSGA-II) to optimise low-power femtosecond laser trepan drilling. They build DNN and random forest models to predict taper and processing time, respectively, and then use NSGA-II to obtain Pareto-optimal process parameters that reduce both taper and drilling time. Similarly, Yan et al.^22^ propose a weighted transferable random forest (WTRF) with incremental structure expansion/reduction for few-shot cross-domain NILM on resource-constrained devices. These methods share with ours the use of optimisation and, in some cases, data-driven surrogate models, but their decision variables are continuous process parameters or model structures, and the optimisation goals are prediction accuracy or processing efficiency.

At the level of stochastic representations and hardware robustness, Chen et al.^23^ study bit-width accumulation in hybrid stochastic numbers (HSNs) and propose a recoding scheme that significantly reduces hardware resources while preserving the intrinsic fault-tolerant properties of stochastic computing. Their work reinforces the role of stochastic representations in achieving numerical robustness under hardware faults.

Compared with these studies, our APAM-IGLLM framework addresses a distinct problem: we do not adapt the structure of a predictive model, nor do we redesign stochastic number formats or optimise physical process parameters. Instead, we regard bit sequences themselves as optimisation variables and employ an evolutionary search guided by a large language model to generate *borderline* random sequences under five strongly coupled statistical tests. The LLM acts as a history-aware controller that dynamically adjusts metric weights and scaling factors in the fitness function based on the evolution of multiple test statistics, turning parameter tuning into a sequential decision process. This optimisation-driven adversarial test data generation for randomness testers is not covered by the above literature.

### Relation to broader optimisation and robustness studies

Taken together, the above works illustrate how optimisation, online adaptation, and robust stochastic methods can enhance performance and reliability in a wide range of scenarios, from physical control and engineering design to anomaly detection, energy scheduling, and cyber defence. Our work is complementary to these studies: instead of optimising system behaviour, structural parameters, or stochastic representations, we optimise over the discrete space of bit sequences to synthesise rare, statistically controlled borderline random sequences. This shift from “optimising systems” to “optimising adversarial test inputs” motivates our algorithmic design, where a genetic algorithm explores the search space and a large language model acts as a meta-level controller that uses short-term memory and reward signals to adapt the optimisation process itself, providing a new tool for fault injection and stress testing of real-time randomness testers.

## Methods

### Motivation

Traditional random number generators (RNGs) produce pseudo-random sequences that exhibit favorable randomness characteristics within certain sequence lengths, making them difficult to meet the requirements of anomalous test cases. Directly generating defective samples by perturbing the 0–1 generation probability is an intuitive approach. To observe the performance of different probability generation scenarios, this study employed a multi-armed bandit method to conduct 3,000 trials with varying 0–1 generation probabilities. Results showed that samples closer to the borderline interval (i.e., those with indicators near the acceptable randomness boundary) achieved higher scores. Notably, no single probability distribution demonstrated a clear advantage; instead, different probabilities induced fluctuations across all statistical test metrics but failed to systematically drive the indicators toward the desired direction.

Let the five test statistics be denoted as the vector

$$X = [X_1, X_2, X_3, X_4, X_5].$$

Under the (idealized) independence assumption of each statistic, their joint rejection region is defined as follows:

1 $$\begin{aligned} X > X_\alpha := \left[ \chi _{1,\alpha }^2,\, \chi _{2,\alpha }^2,\, \chi _{2^{m}-1,\alpha }^2,\, N^{-1}(1-\alpha / 2),\, \chi _{2k-2,\alpha }^2 \right] . \end{aligned}$$

Inter-test correlations further constrain the theoretical probabilities under the independence assumption. Statistical metrics from each test depend on shared underlying parameters (e.g., $n_0, n_1, n_{ij}$, etc.), such that adjusting one parameter influences multiple statistical metrics. Generating samples in a globally borderline state or with individual indicators in a borderline state therefore requires not only satisfying multiple statistical constraints but also overcoming the challenges of nonlinear correlations between tests and the complexity of high-dimensional solution spaces.

The borderline region of interest can be described as a hyper-rectangle

$$\left( X_\alpha , 1.3X_\alpha \right] ,$$

where the interval (1, 1.3] (or $(0.9,\infty )$ for the autocorrelation test) corresponds to “slightly unqualified” or “borderline” randomness for each metric. Assuming the sequence length is $O(2^n)$, the parameter space dimension is $O(2^n)$, making the direct application of traditional brute-force search infeasible. The nonlinear correlations among dimensions (statistical metrics) result in an extremely narrow feasible solution space, where the probability of generating borderline-defect samples can be written as:

2 $$\begin{aligned} \textrm{P} = \int _{X_\alpha }^{1.3 X_\alpha } \cdots \int _{X_\alpha }^{1.3 X_\alpha } f_{X_1 X_2 X_3 X_4 X_5}(x_1,x_2,x_3,x_4,x_5) \, dx_1 dx_2 dx_3 dx_4 dx_5. \end{aligned}$$

The study conducted approximate solutions using 1 million Monte Carlo simulations, with the probabilities of various defect patterns presented in Tables 2 and 3. It is evident that directly addressing this issue poses significant challenges.

Beyond probabilistic perturbation, this study also explored other potential methods, such as deep generative models (e.g., Conditional Generative Adversarial Networks, Conditional GANs), which have significantly advanced the generation of samples with complex distributions^42–46^. However, experimental and analytical findings reveal that such methods struggle to flexibly control the length of generated sequences and incur high training costs, thus rendering them unsuitable for the current task.

### Borderline defect sample test case generation method

**Fig. 1:**
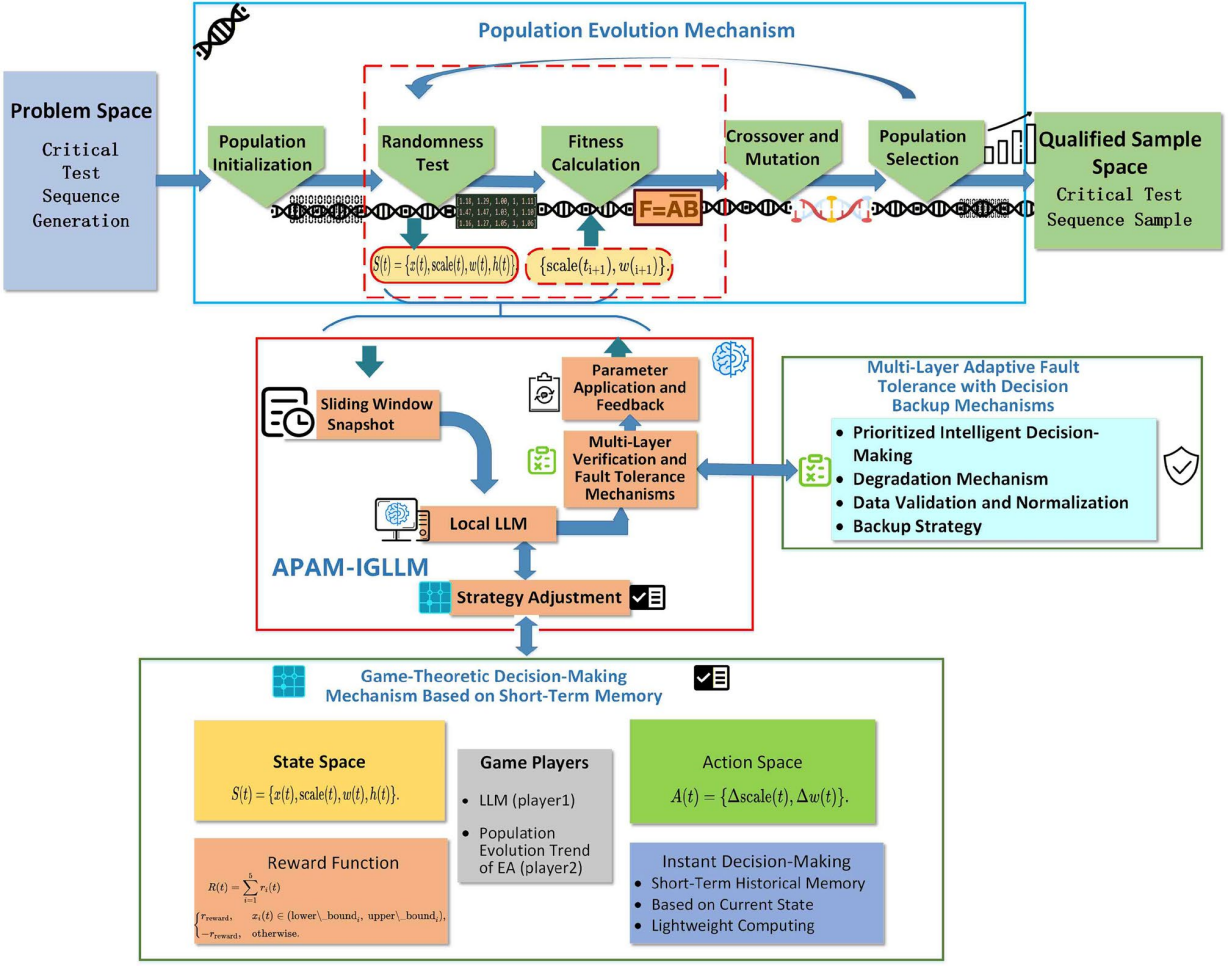
Overall architecture of the proposed APAM-IGLLM based evolutionary test-case generation framework.

This study transforms the sequence generation problem into a multi-constrained optimization problem in a high-dimensional binary space and proposes a controllable sequence generation framework that combines an evolutionary algorithm (EA) with large language models (LLMs): the Algorithm Parameter Adjustment Mechanism Based on Intelligent Game with Large Language Models (APAM-IGLLM). Figure 1 presents the overall architecture of the proposed framework.

Specifically, the method represents target samples as 0–1 sequences aligned with the required length of random sequences. Through dynamically regulated population evolution guided by an LLM-based controller, the population is progressively steered toward the target borderline region. Unlike conventional approaches, the framework directly leverages a randomness test algorithm to generate a normalized five-metric vector (distribution values) for each sequence. These values are scaled by test-specific scaling factors and combined with dynamically adjusted weight coefficients to compute fitness values and survival probabilities, thereby balancing multiple randomness constraints and search efficiency.Compared with traditional evolutionary algorithms using static or rule-based penalties, this LLM-based controller provides a history-aware, multi-metric adaptation mechanism that better explores the extremely thin joint borderline region defined by the five randomness tests.

When generating large-scale sequences (e.g., 102,400 bits), static weight coefficients and scaling factors can lead the randomness metrics to fall into local optima, making it challenging to generate sequences that meet the borderline requirements. Single-time manual adjustment of these parameters can locally improve certain metrics, but is insufficient for high-dimensional, long-sequence tasks. Therefore, this work introduces a systematic dynamic adjustment mechanism: at each evolutionary generation, the scaling factors and weight coefficients are updated based on the population’s multi-metric statistics, target intervals, and recent evolutionary trends.

To avoid premature convergence and to adapt to changes in the optimization scale, the proposed APAM-IGLLM uses an LLM as an intelligent decision maker. The LLM receives a compact description of the current state (including metric distributions, success proportion, and historical records) and outputs refined parameter suggestions (scaling factors and weights) for the next generation, according to game-theoretic rules. This ensures a balanced optimization across multiple tests while maintaining computational efficiency. The design of the Game-Theoretic Decision-Making Mechanism Based on Short-Term Memory is described in detail in section "Population evolution and LLM-based parameter adjustment".

To prevent intelligent decision-making failures, an additional safety fault-tolerance mechanism–rule-based Multi-Layer Adaptive Fault Tolerance with Decision Backup Mechanisms–is incorporated. Its primary functions include:

- prioritizing LLM-based Intelligent Decision-Making for parameter updates;
- when the LLM response is invalid or unavailable, automatically switching to rule-based adjustments to generate alternative decisions;
- performing consistency checks, range validation, and normalization to ensure that the returned outputs meet required specifications.

During each generation of evolution, samples meeting the borderline criteria are screened and stored in real time.

### Framework and analysis

#### Population encoding and decoding

The population initialization module generates random and diverse initial sequences, with each sample represented as a binary sequence of specified length. Let the population size be *P* and the sample length be *N*. The initial encoding can be mathematically expressed as:

$$\text {Pop} = \left\{ S_i \mid S_i \in \{0,1\}^{N},\, i=1,2,\ldots ,P \right\} ,$$

where $S_i$ denotes the *i*-th sample sequence.

The decoding module does not perform base conversion. Instead, it maps each binary sequence $S_i$ to a normalized result vector composed of the statistical results of the five randomness tests:

$$\{ \text {Frequency Test},\ \text {Pair Test},\ \text {Poker Test},\ \text {Autocorrelation Test},\ \text {Run Test} \}.$$

The result of each test is used to evaluate a specific randomness indicator of the sample. Detailed statistical formulas and normalization processes are presented in Table 1. Finally, the decoding process yields the statistical result vector

$$S_i \rightarrow \{ f_1(X_1), f_2(X_2), f_3(X_3), f_4(X_4), f_5(X_5) \}.$$

A sample is considered *borderline qualified* if its five normalized metrics $(X_1,X_2,X_3,X_4,X_5)$ satisfy

3 $$\begin{aligned} \begin{aligned} 1&< X_j \le 1.3, & j \in \{1,2,3,5\},\\ X_4&> 0.9. & \end{aligned} \end{aligned}$$

These conditions are consistent with the scaling and reward design used in the optimization process and with the implementation in the experimental code.

#### Fitness function and dynamic penalty design

The fitness function is employed to evaluate sample quality and to guide the population toward the borderline region defined in (3). By scaling the results of the five randomness tests and assigning weights, the fitness value of each sample $S_i$ is computed as:

4 $$\begin{aligned} F(S_i) = \sum _{j=1}^5 \omega _j \cdot \operatorname {Penalty}(\tilde{X}_j), \end{aligned}$$

where $\omega _j$ represents the weight of the *j*-th test and $\tilde{X}_j$ denotes the scaled metric for that test.

To amplify deviations from the target interval, the metrics are scaled as follows. For $j \in \{1,2,3,5\}$, when $X_j < 1$,

5 $$\begin{aligned} \tilde{X}_j = k_j \cdot \frac{1}{\max (X_j, 10^{-4})}, \end{aligned}$$

otherwise $\tilde{X}_j = X_j$. This design increases the contribution of clearly unqualified samples (with $X_j < 1$) to the penalty term, using the scaling factor $k_j$ as a tunable sensitivity coefficient. For the autocorrelation test ($j=4$), due to its distinct target range, a different transformation is used:

6 $$\begin{aligned} \tilde{X}_4 = \frac{X_4}{4} + k_4. \end{aligned}$$

The penalty term $\operatorname {Penalty}(\tilde{X}_j)$ is defined as:

7 $$\begin{aligned} \operatorname {Penalty}(\tilde{X}_j) = {\left\{ \begin{array}{ll} \displaystyle \frac{1}{\tilde{X}_j}, & j \in \{1,2,3,5\},\\ \displaystyle \tilde{X}_4, & j = 4. \end{array}\right. } \end{aligned}$$

In other words, for Frequency, Pair, Poker and Run tests, smaller (worse) values yield larger penalties, whereas for Autocorrelation the transformed value itself is used. In practice, the fitness values are further shifted and scaled to ensure numerical stability and to avoid zero fitness in selection:

$$\text {fitness}(S_i) = F(S_i) - \min _{k} F(S_k) + \varepsilon ,$$

with a small $$\varepsilon >0$$.

The weight coefficients $\omega _j$ and scaling factors $k_j$ are dynamically adjusted based on the evolutionary trends of the population. Intuitively, metrics that deviate more from the target range receive higher priority (larger effective penalty) in the subsequent generations, either by increasing their scaling factors $k_j$ or by adjusting $\omega _j$ to emphasize or de-emphasize their contribution to the fitness.

#### Population evolution and LLM-based parameter adjustment

Population evolution is implemented through standard evolutionary operations: selection, crossover, and mutation. At generation *t*, the current population $\text {Pop}(t)$ is evaluated by decoding each sequence and computing its fitness through (4). The EA then selects individuals with probability proportional to their fitness, applies crossover to generate offspring, and introduces random mutations to maintain diversity.

However, the performance of this evolutionary process critically depends on the proper choice of the scaling factors $k_j$ and weights $\omega _j$. Static or manually tuned parameters are prone to trapping the population in local optima, especially for long sequences and narrow borderline regions. To address this issue, this study proposes APAM-IGLLM, which treats the LLM as an optimization regulator that adaptively adjusts $(k_j,\omega _j)$ based on a game-theoretic view of the interaction between the EA and the LLM.

**State Space.** At the *t*-th generation, the system state *S*(*t*) is defined as:

8 $$\begin{aligned} S(t) = \{ x(t), \text {scale}(t), w(t), h(t) \}. \end{aligned}$$

Here, *x*(*t*) denotes the metrics of the current population (e.g., the matrix of five test values for all individuals, together with aggregated statistics such as the number of borderline qualified samples); $\text {scale}(t)$ denotes the current scaling factors $k_j$; *w*(*t*) denotes the current weight coefficients $\omega _j$; and *h*(*t*) is a dynamic short-term history window containing the scaling factors, weight coefficients, and key test statistics of the most recent *k* generations.

**Action Space.** The system’s action *A*(*t*) refers to the adjustment of scaling factors and weights for the next generation:

9 $$\begin{aligned} A(t) = \{\Delta \text {scale}(t), \Delta w(t)\}. \end{aligned}$$

Here, $\Delta \text {scale}(t)$ represents the change applied to the scaling factors (e.g., multiplicative increases or decreases within a bounded range), and $\Delta w(t)$ represents the change applied to the weight coefficients, which are subsequently re-normalized to satisfy $\sum _j \omega _j = 1$.

**Reward Function.** The reward function *R*(*t*) reflects the quality of the current generation with respect to the target borderline region:

10 $$\begin{aligned} R(t) = \sum _{i=1}^5 r_i(t), \end{aligned}$$

where the per-metric reward $r_i(t)$ is defined as

11 $$\begin{aligned} r_i(t) = {\left\{ \begin{array}{ll} r_{\text {reward}}, & x_i(t) \in \left( \text {lower\_bound}_i,\ \text {upper\_bound}_i\right) ,\\ -r_{\text {reward}}, & \text {otherwise}. \end{array}\right. } \end{aligned}$$

The values $r_{\text {reward}}$ and $-r_{\text {reward}}$ represent reward and penalty, respectively, indicating whether each metric lies within its target interval (e.g., (1, 1.3] for Frequency, Pair, Poker, and Run tests, and $(0.9,\infty )$ for the Autocorrelation test). The overall optimization objective of the framework is to maximize the cumulative reward:

12 $$\begin{aligned} \max \sum _{t=1}^{T} R(t), \end{aligned}$$

where *T* denotes the total number of generations. This objective is equivalent to maximizing the number and stability of borderline qualified samples over the entire evolutionary process.

**Game-Theoretic Interpretation.** The interaction between the EA and the LLM can be viewed as a repeated game with short-term memory. At each generation:

- The EA (player 1) generates a population under the current parameters $(\text {scale}(t), w(t))$ and produces the observed metrics *x*(*t*).
- The LLM (player 2) observes the summarized state *S*(*t*), including *x*(*t*) and the recent history *h*(*t*), and chooses an action *A*(*t*) by proposing updated scaling factors and weights for the next generation.

**Fig. 2:**
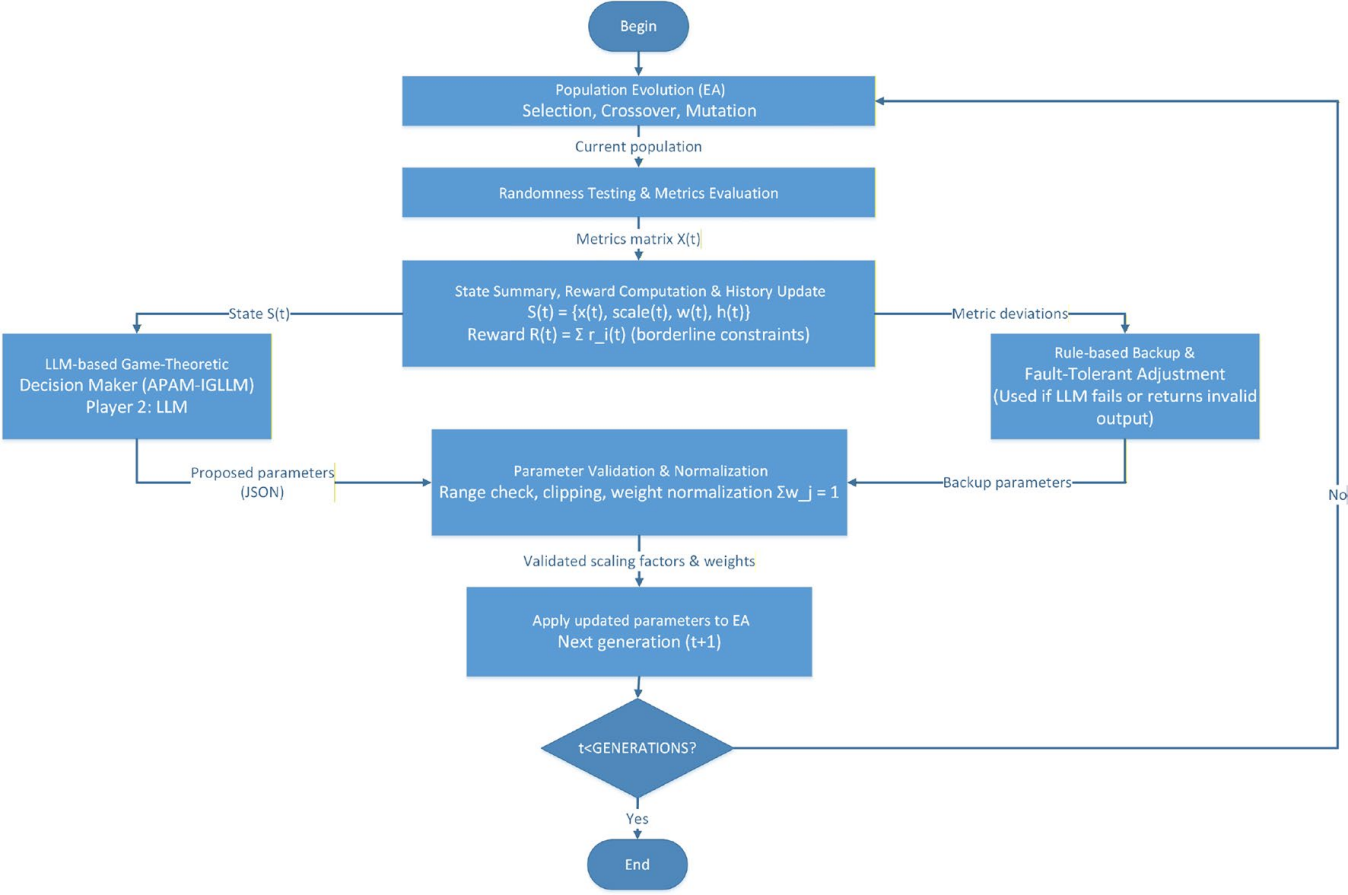
Game-theoretic interaction between the evolutionary algorithm (EA) and the LLM-based parameter adjustment mechanism (APAM-IGLLM). At each generation, the EA evaluates the current population and sends a summarized state, including metrics and short-term history, to the LLM. The LLM acts as a high-level decision maker, proposing updated scaling factors and weights according to a repeated-game reward signal that reflects constraint satisfaction in the borderline region. A fault-tolerant rule-based backup module ensures stability and continued optimization when the LLM response is unavailable or invalid. The updated parameters are then fed back to the EA to guide the next generation of evolution, forming a closed-loop, game-theoretic optimization process.

When the proportion of borderline qualified samples and the cumulative reward *R*(*t*) are low, the LLM tends to propose more aggressive parameter changes (e.g., larger adjustments to scaling factors or reallocation of weights toward the most deviating metrics). When the reward is high, the LLM proposals become more conservative, stabilizing the search around promising borderline solutions. This leader–follower, repeated-game perspective clarifies how the LLM–EA interaction improves constraint handling and avoids premature convergence, as illustrated in Fig. 2.

**Decision Process.** At each generation *t*, the decision process proceeds as follows:

- *Evaluation of metric deviation:* The framework evaluates the deviation of each metric from its target range, both at the individual level and in terms of population aggregates (e.g., mean, variance, and proportion of qualified samples).
- *Historical trend analysis:* Using *h*(*t*), the framework analyzes the trends of metric changes and reward signals over the most recent *k* generations to detect stagnation or instability.
- *Action generation:* Based on the current state and historical trends, the LLM generates an action *A*(*t*), i.e., adjustments to scaling factors and weight coefficients, in JSON format.
- *Feedback and update:* The framework applies the validated adjustments, computes the new metrics and reward $R(t+1)$, and updates the historical record $h(t+1)$.

In practical implementation, the LLM receives a structured prompt summarizing the current scaling factors, weights, success counts, metric distributions, and the last *k* generations’ records. It returns a JSON object containing proposed new scaling factors and weights, which are then checked for validity (range constraints) and re-normalized before being applied.

**Fault-Tolerant Rule-Based Backup.**

If the LLM call fails or returns an invalid response, the framework falls back to a rule-based adjustment mechanism. This backup mechanism adjusts weights and scaling factors according to measurable metric deviations. For example, for each metric *i*:

- if the mean metric value is below the lower bound, the corresponding scaling factor is multiplied by a reward factor (e.g., 1.05);
- if the mean value is above the upper bound, the scaling factor is multiplied by a penalty factor (e.g., 0.95);
- weights are adjusted proportionally to normalized deviations and then re-normalized to ensure $\sum _i \omega _i = 1$.

This rule-based module ensures that the optimization process can continue and remain stable even when intelligent decision-making is temporarily unavailable.

#### APAM-IGLLM parameter adjustment algorithm

The overall parameter adjustment procedure combining the LLM-based game-theoretic mechanism and the rule-based backup can be summarized as Algorithm 1. This algorithm corresponds to the core implementation used in our experiments.In addition to the randomness-testing task, we further evaluate the optimisation capability of APAM-IGLLM on the CEC 2017 benchmark functions and compare it with several classical metaheuristic algorithms in section “Borderline sample generation for five randomness metrics”.

**Algorithm 1:**
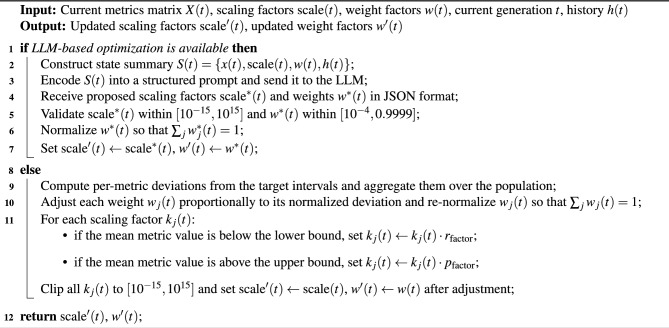
APAM-IGLLM: Scaling and Weight Factor Adjustment

#### Complexity and convergence discussion

The system maintains five-generation historical adjustment records of scaling factors $s$, weight coefficients $w$, and rewards $r$, denoted as $\left( s^{(t-4)}, w^{(t-4)}, r^{(t-4)}\right)$. These records are fed into a predictive model integrated with a large language model (LLM). The LLM generates parameter adjustment strategies $\Delta s$ and $\Delta w$ to maximise the cumulative reward $R^t = \sum _i r_i^t$. This reward-driven mechanism effectively turns a high-dimensional nonlinear correlation problem into a dynamic game involving the LLM, which leverages its semantic understanding of short-term historical trends to indirectly optimise the mapping between parameters and metrics.

From a continuous-time perspective, the convergence behaviour of traditional evolutionary methods can be informally written as $\frac{d X_i}{d t} = -\nabla F(X)$. In contrast, the proposed method actively introduces perturbation terms through $\Delta s$ and $\Delta w$:

$$\frac{d X_i}{d t} = -\nabla F(X) + \sigma \bigl (s^{(t)}, s^{(t-1)}\bigr ) + \tau \bigl (w^{(t)}, w^{(t-1)}\bigr ),$$

where $\sigma (\cdot )$ and $\tau (\cdot )$ represent, respectively, the step function for scaling-factor adjustments and the smoothing term for weight-coefficient updates. These perturbations disrupt the inherent structure of the covariance matrix among metrics and gradually attenuate correlations in non-target directions during intergenerational evolution. As a result, the effective probability density of the feasible solution space is expanded, alleviating the super-exponential growth of constraint complexity under the independence assumption.

It provides an intuitive view that the dynamic perturbations induced by APAM-IGLLM heuristically reduce the search difficulty from an astronomical combinatorial space $\mathcal {O}\!\left( 2^{102400}\right)$ to tractable gradient-like updates $\mathcal {O}(T \cdot P \cdot K)$, where $T$ is the number of generations, $P$ the population size, and $K$ the number of metrics. This interpretation is consistent with the empirical convergence behaviour observed in our experiments.

## Results

To evaluate the effectiveness of the APAM-IGLLM mechanism, we conduct experiments to address the following questions: How does APAM-IGLLM perform compared with classical optimisation algorithms? How effective is APAM-IGLLM when generating samples in which all metrics are simultaneously at their borderline values versus samples that are borderline with respect to a single metric? Is the APAM-IGLLM mechanism genuinely effective for the task of generating borderline random samples? How do other comparable evolutionary algorithms perform on this task? Finally, how do the generated borderline random samples behave under practical randomness testing?This chapter conducts experiments around the above questions and analyses the corresponding results.

### Comparative experiments on standard benchmark functions

Although the GAPAM-IGLLM method is primarily designed for solving borderline random sequences in the borderline random sample generation task rather than for general optimisation, we still evaluate its performance and effectiveness on the CEC 2017 benchmark test suite. Since GAPAM-IGLLM is mainly designed for high-dimensional long-sequence problems, the dimensionality in all tests is fixed at 100, and each case is evolved for 500 generations. In the original GAPAM-IGLLM, the multi-indicator weights and scaling factors of the evolutionary algorithm (EA) are adjusted online by a large language model (LLM) according to historical data. This process is essentially equivalent to dynamically reshaping the evaluation function *F*.

To ensure the fairness and reproducibility of benchmark performance comparisons, this experiment simulates the Large Language Model (LLM)-based adjustment mechanism through a hard-coded approach that incorporates historical records for adaptive factor adjustment. All algorithms are compared on the same CEC2017 objective functions; GAPAM-IGLLM only adjusts the search behavior of the EA internally and no longer modifies the objective function itself. From a theoretical perspective, this version can be regarded as a “performance-weakened” variant of the original GAPAM-IGLLM, but it does not rely on any external model and is therefore more suitable as a general optimization algorithm for fair benchmarking against other methods. As baselines, we select several representative evolutionary and swarm intelligence algorithms, including the Genetic Algorithm (GA)^47^, Differential Evolution (DE)^48^, Black-Winged Kite Algorithm (BKA)^49^, Particle Swarm Optimization (PSO)^50^, Harris Hawks Optimization (HHO)^51^, Dung Beetle Optimizer (DBO)^52^ and Coyote Optimization Algorithm (COA)^53^.

**Fig. 3:**
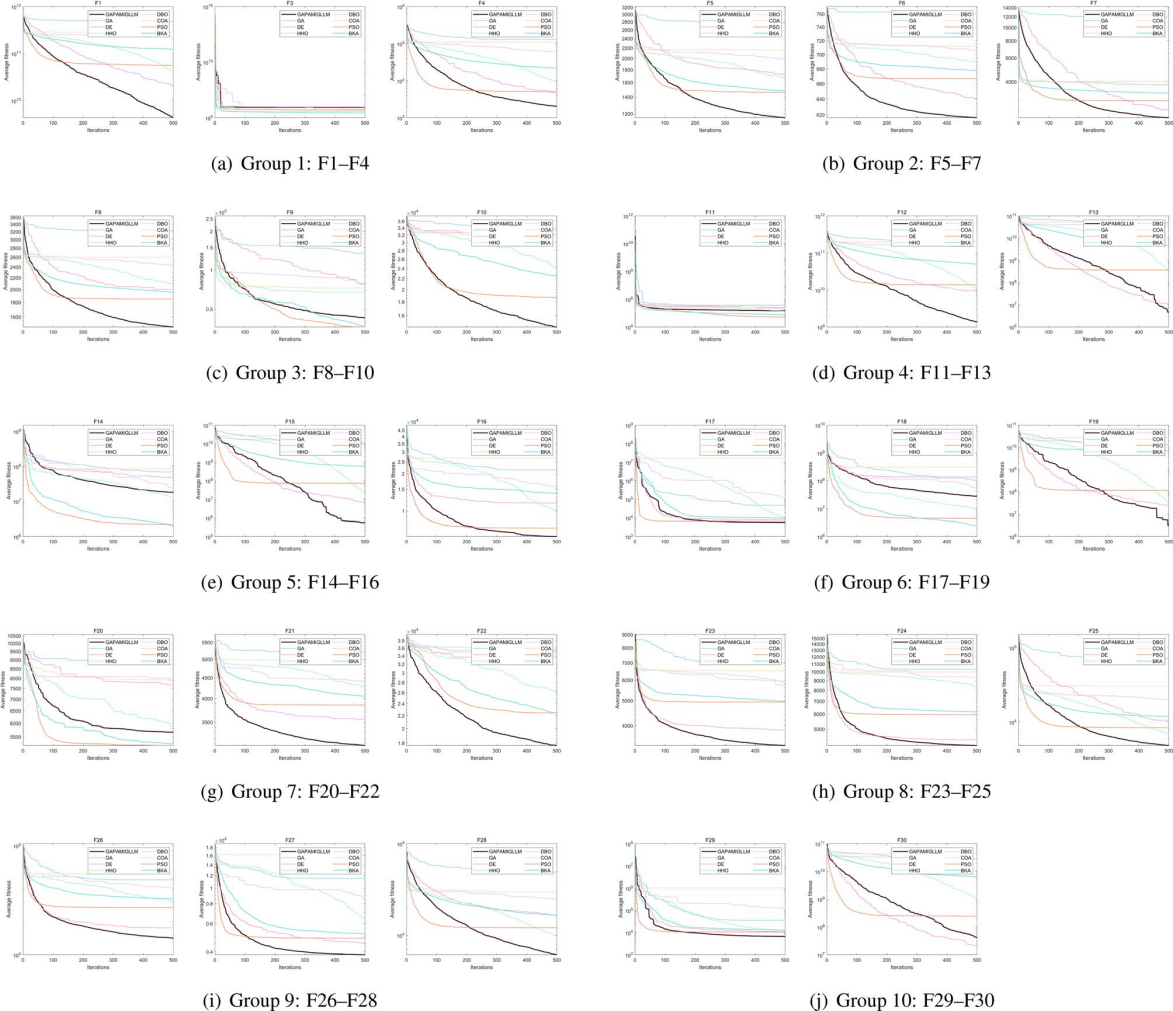
Convergence graphs of compared algorithms on benchmark functions F1–F30.

**Fig. 4:**
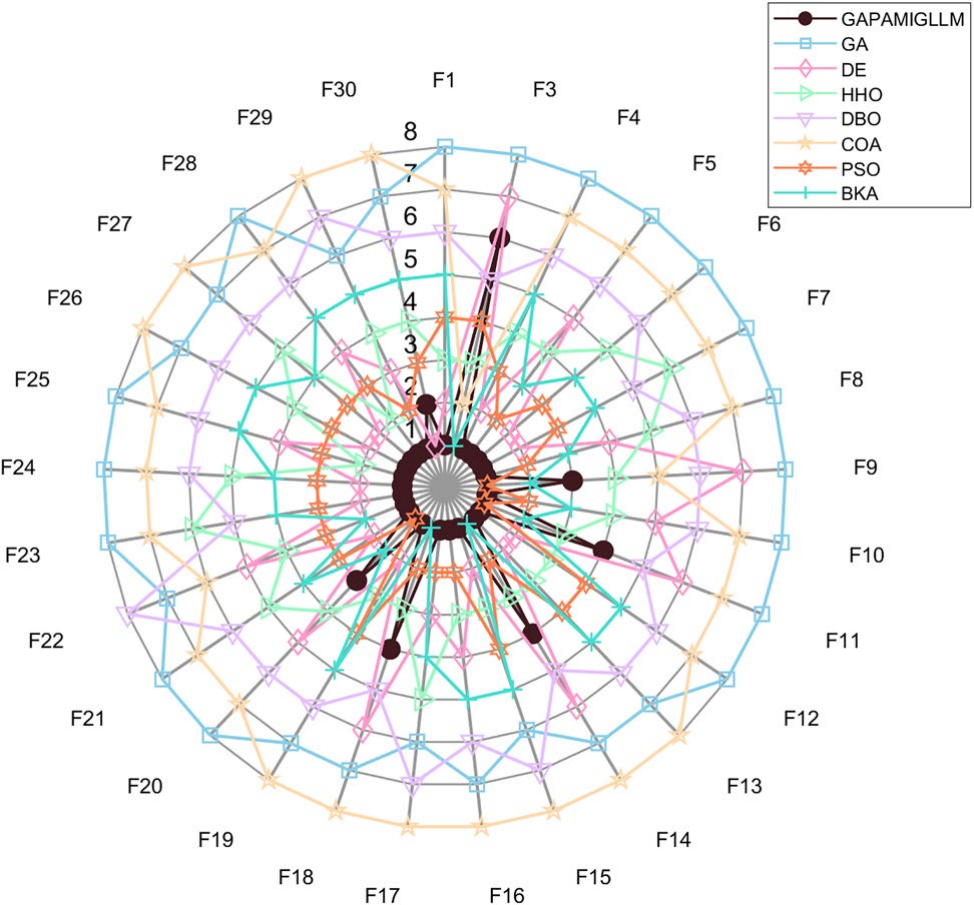
Radar plot of the compared algorithms on benchmark functions F1–F30, where the ranking is based on the best values and variances; a smaller enclosed area indicates better performance.

Figure 3 illustrates the convergence behavior of the compared algorithms on benchmark functions F1–F30, while Fig. 4 presents the corresponding best objective values (solution accuracy) achieved by each algorithm.In terms of results, GAPAM-IGLLM exhibits clearly superior convergence speed and final accuracy compared with the conventional GA on the vast majority of test functions. This advantage is particularly evident on multi-modal and composition functions (F9–F10, F16–F17, F22–F28), where the standard GA often suffers from premature convergence in the middle stages, while GAPAM-IGLLM continues to make progress. This demonstrates that the multi-factor state feedback provides a substantial improvement in balancing exploration and exploitation for GA. DE, HHO, and DBO achieve more prominent performance on a few specific problems (e.g., certain ill-conditioned or hybrid functions); however, overall GAPAM-IGLLM ranks in the first or second tier on most functions. Its performance is comparable to these well-engineered continuous optimizers and, at the same time, it avoids the apparent failures that some algorithms encounter on part of the test set.

Swarm intelligence algorithms such as PSO, COA, and BKA usually show a very fast decrease in the early iterations, but their convergence curves often become almost flat after around 100 generations. In contrast, GAPAM-IGLLM behaves more like a robust optimizer: its early convergence is slightly slower, but it continues to improve in the later stages, and its convergence curves are smoother overall, indicating better adaptability to complex landscapes.

Under this conservative setting, GAPAM-IGLLM still achieves overall leading or highly competitive performance on the CEC2017 100-dimensional benchmark. This implies that the original GA+LLM idea is not only effective for specific engineering tasks, but that the rule-based, “weakened” variant—i.e., the multi-factor adaptive EA framework itself—also possesses strong general optimization capability. These results provide solid evidence for the generality and transferability of the proposed framework.

### Borderline sample generation for five randomness metrics

First, different local large language models (LLMs) are utilized to generate samples of varying lengths, aiming to validate the effectiveness of the proposed method.

**Fig. 5:**
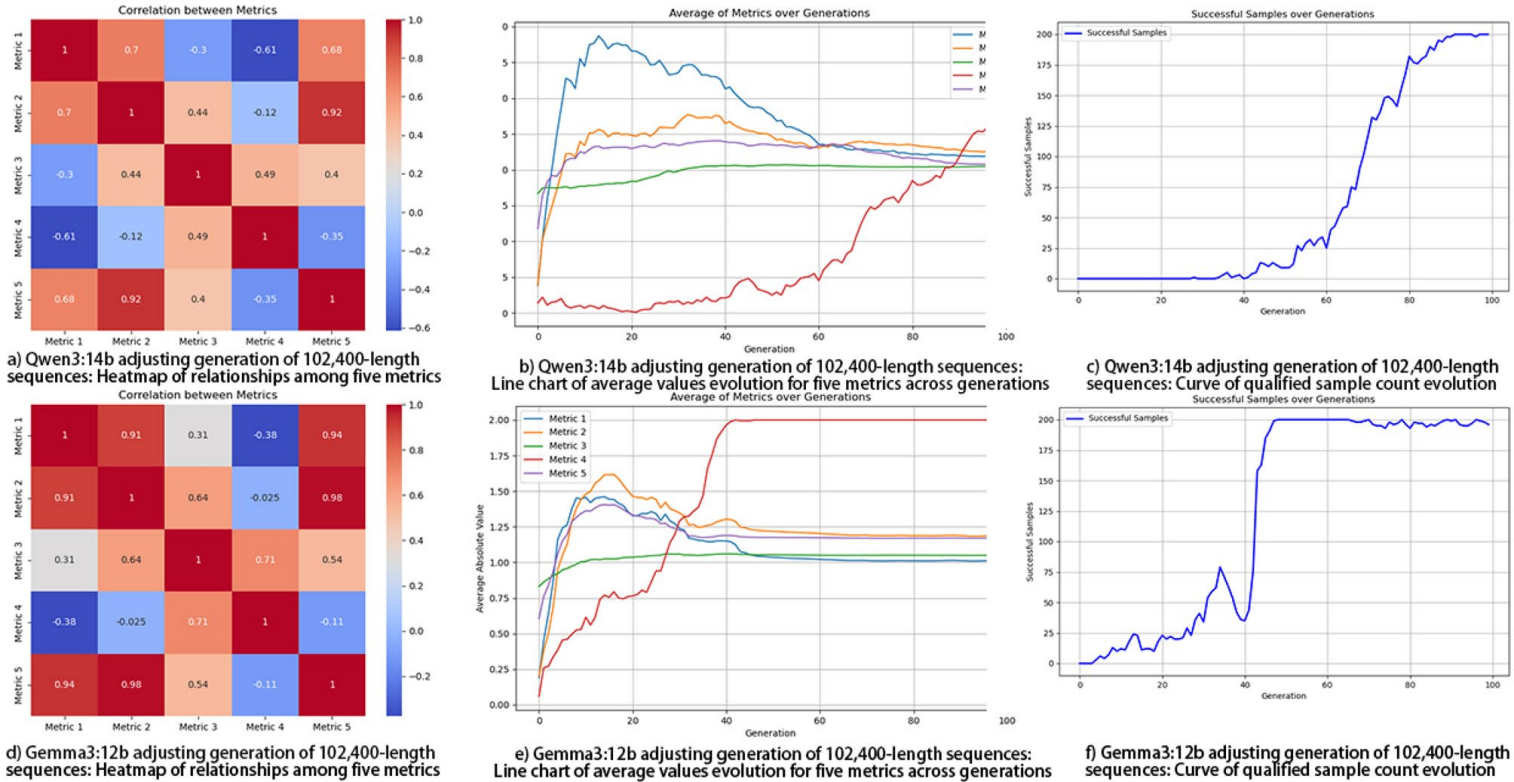
Performance of borderline Sample Generation for Five Test Metrics. As shown in the heatmap, significant correlations indeed exist among these five indicators. Subplots (**b**) and (**e**) in the above figure depict the average variation of the five test indicator values across generations. Among them, the red line represents the normalized indicator of the autocorrelation test; due to its distinct counting method compared to other indicators, higher values of this line indicate stronger alignment with borderline requirements, while the other four lines should converge within the target range. Experimental results show that the adjustments of both models tend to converge during the middle and late stages of evolution.

The initial values of the scaling factors for the five metrics are: 10,000, 10,000, 10,000, 0.001, and 100,000, respectively, while the initial values of all weight coefficients are 0.2. Due to the significant disparities among the scaling factors, a logarithmic scale is also used for easier observation. Figure 5 shows the relevant experimental results.

### Single-defect pattern sample generation

**Fig. 6:**
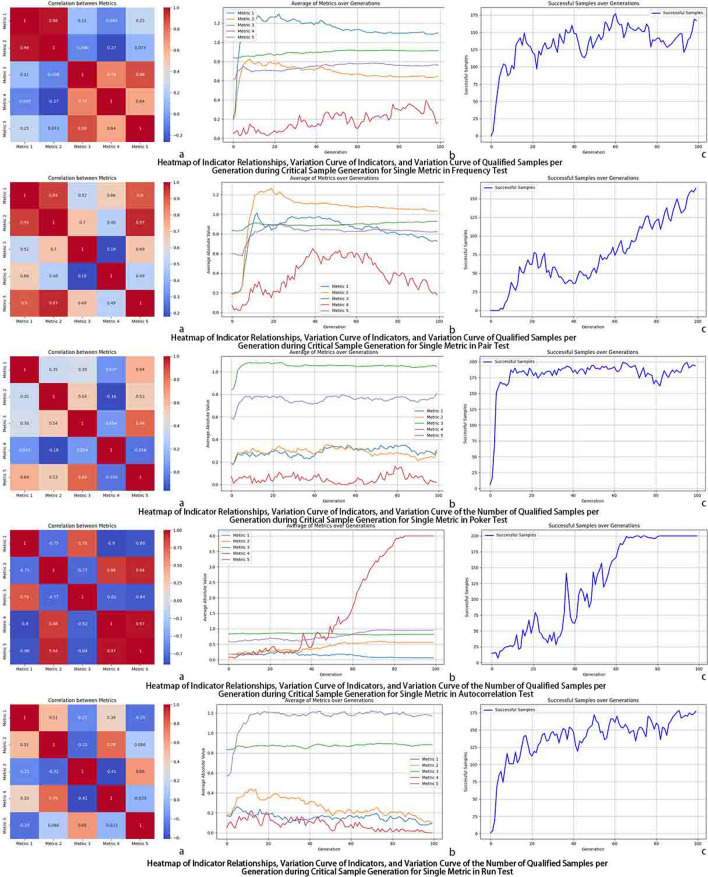
Performance of single-type defect sample generation.

Since the generation probability of single-defect samples is significantly higher than that of samples with five borderline metrics, as shown in Fig. 6, the required metrics often achieve convergence rapidly.

### Ablation and comparative studies of the APAM-IGLLM mechanism

To further substantiate the convergence improvement and reduced search complexity, we conduct ablation and comparative experiments. In the ablation setting, the APAM-IGLLM mechanism is disabled and the EA runs with several static parameter configurations. We then compare these variants with Differential Evolution (DE) and the Non-dominated Sorting Genetic Algorithm II (NSGA-II) on the same borderline-sample generation task.

In this task, five scaling factors and five weight coefficients transform and weight the five randomness metrics in the fitness function. With APAM-IGLLM enabled, these ten parameters are dynamically adjusted during evolution; in the ablation experiments, they are kept fixed to examine their influence. Four groups of fixed settings are tested. The first adopts an approximately uniform configuration:

$$(10{,}000, 10{,}000, 10{,}000, 0.01, 100{,}000, 0.20, 0.20, 0.20, 0.20, 0.20),$$

where the fourth scaling factor is smaller than 1 because of its different range, and the fifth corresponds to an empirical value from earlier short-sequence experiments. The other three groups bias the parameters towards different metrics, e.g.

$$(150{,}000, 10{,}000, 1{,}000, 0.05, 100{,}000, 0.22, 0.19, 0.19, 0.21, 0.19),$$

$$(100{,}000, 10{,}000, 10{,}000, 0.01, 100{,}000, 0.24, 0.19, 0.19, 0.19, 0.19),$$

and

$$(15{,}000, 11{,}000, 0.08, 100{,}000, 0.22, 0.18, 0.19, 0.23, 0.18),$$

which, for instance, up-weight Metrics 1 and 4 while down-weighting Metrics 2 and 5.

**Fig. 7:**
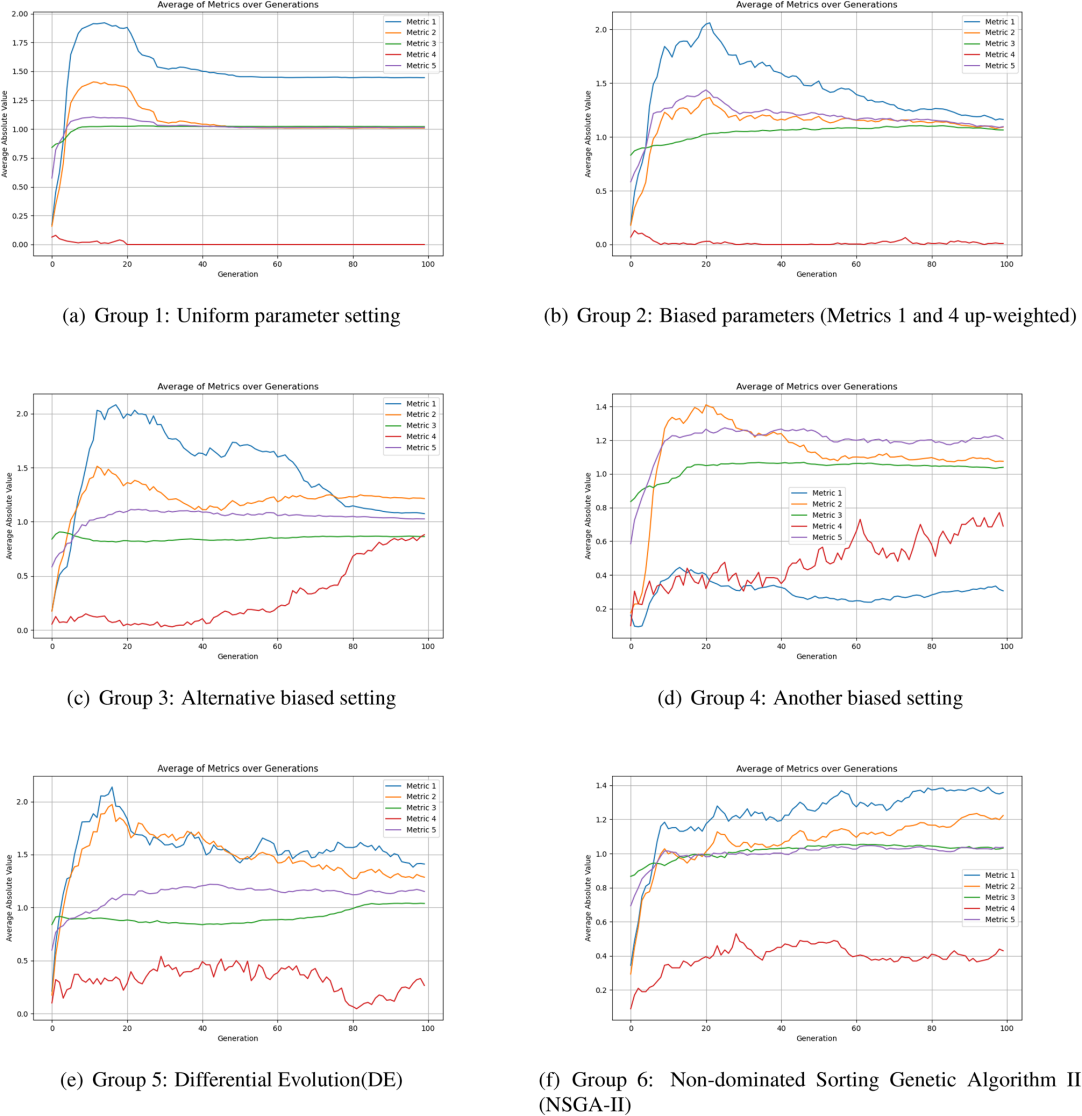
Convergence of the five randomness metrics under four fixed parameter settings without APAM-IGLLM, and under Differential Evolution (DE) and Non-dominated Sorting Genetic Algorithm II (NSGA-II). None of the static configurations is able to drive all five metrics into the borderline region simultaneously, which highlights the necessity of dynamic parameter adjustment. Compared with DE and NSGA-II, APAM-IGLLM still achieves the best overall convergence behaviour of the five metrics on this task.

For long-sequence generation, these static settings are unable to tune the metrics with sufficient precision: none of the four groups produces any sample that simultaneously satisfies all five borderline criteria. From the convergence curves in Fig. 7a–d, at least one metric remains outside the target interval in every case–for example, Metrics 1 and 4 never reach 1.3 and 1.0 in Group 1; in Group 3, Metrics 1 and 4 approach the target interval but Metric 3 always stays below 1; and in Group 4, Metrics 1 and 4 oscillate on the left side of the admissible range. These results confirm that the ten parameters strongly affect metric convergence, but manually designed fixed combinations cannot balance all metrics at once and may improve one metric at the expense of others.

In addition to static-parameter baselines, we include DE and NSGA-II as representative evolutionary algorithms. For DE, real-valued differences are replaced by bitwise XOR operations on binary strings, while the original mutation scheme is retained. For NSGA-II, individuals are encoded as binary sequences. For each individual, the randomness-testing module computes five statistics; the absolute deviation of each statistic from its target borderline value is treated as one objective, yielding a five-dimensional multi-objective problem. We employ the NSGA-II implementation in DEAP. By combining parent and offspring populations, performing non-dominated sorting and crowding-distance selection, the algorithm gradually drives the population towards the borderline region of each test while preserving diversity. The final Pareto-optimal individuals correspond to samples that are close to the critical boundaries under all five tests.

Figure 7 and Table 2 summarise the results. All four static parameter settings fail to generate any fully qualified sample, and their median and mean values remain far from the desired borderline intervals. DE and NSGA-II improve upon these baselines and can produce a small number of qualified samples (63 and 78, respectively), but both still rely on fixed parameter structures: DE uses a static differential strategy, and NSGA-II focuses on balancing deviations and maintaining diversity on the Pareto front rather than adaptively pushing the population deep into the narrow feasible region.

**Fig. 8:**
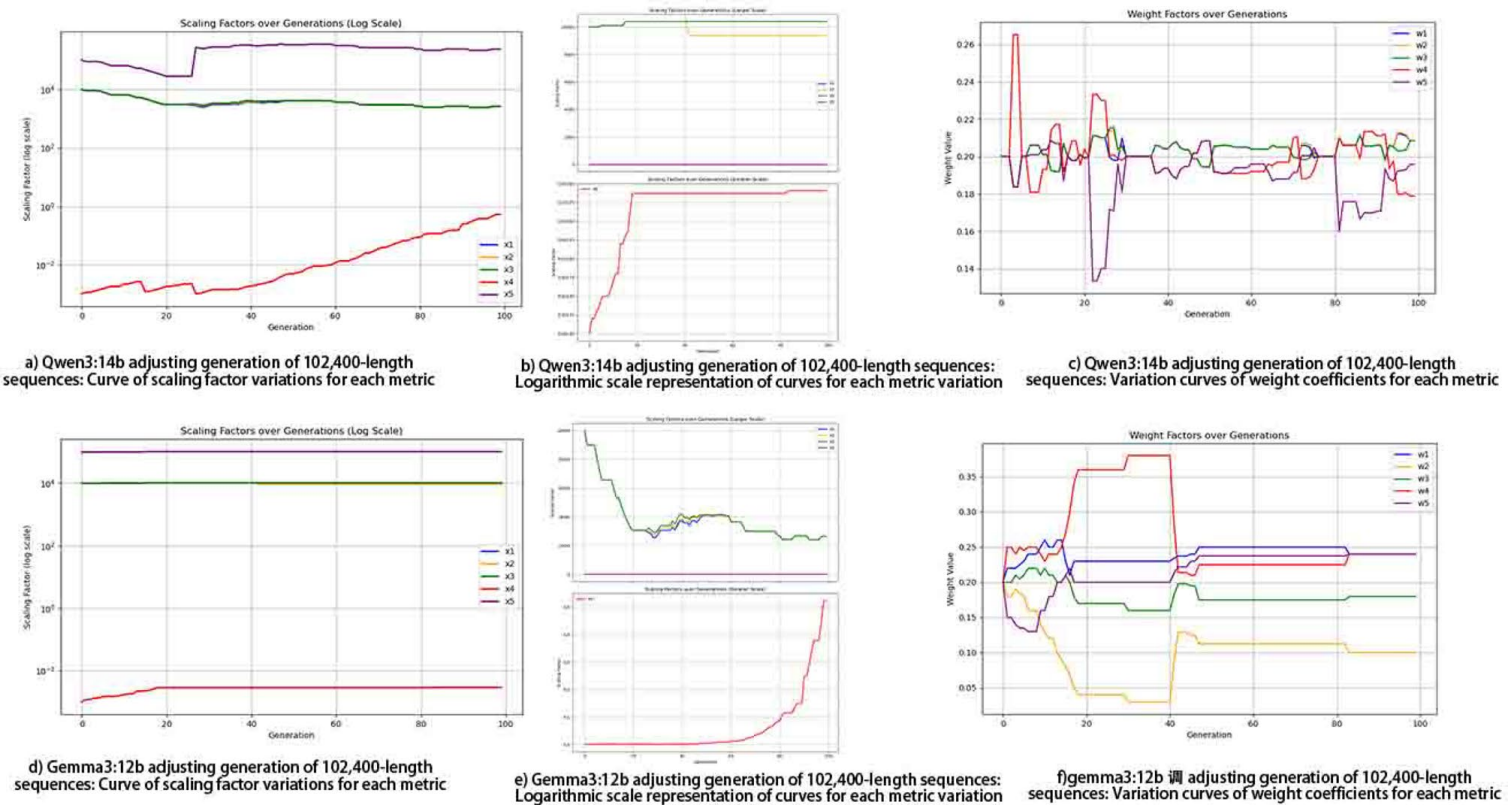
Evolution of scaling factors and weight coefficients under APAM-/IGLLM when generating 102,400-bit borderline sequences. The top row corresponds to the Qwen3:14b-based controller, and the bottom row to the Gemma3:12b-based controller. Subplots (**a**) and (**d**) show the trajectories of the five scaling factors on a logarithmic scale; (**b**) and (**e**) provide zoomed-in views of the variations for each metric; (**c**) and (**f**) present the evolution of the five weight coefficients. As the five randomness metrics gradually converge towards their target borderline intervals, the scaling factors and weights also tend to stabilise, indicating that the LLM-based controller learns a consistent adjustment pattern and that the dynamic parameter adaptation mechanism is effective.

In contrast, APAM-IGLLM consistently generates a large number of borderline-qualified sequences (8,191 samples in Table 2) within a comparable number of generations. Figure 5 shows that Metrics 1, 2, 3 and 5 converge into the borderline interval, while Metric 4 increases steadily, leading to a high success rate. Figure 8 further illustrates that the associated scaling factors and weights undergo large adjustments in the early generations and gradually stabilise as the metrics approach their targets, indicating that the LLM-based controller has learned a consistent and effective adjustment pattern. Overall, these findings demonstrate that dynamic parameter adaptation is crucial for this high-dimensional, multi-test constrained setting and that APAM-IGLLM achieves the best overall convergence behaviour among all compared methods.

**Table 2:** All four static parameter settings fail to produce samples that simultaneously satisfy the five borderline conditions, and their metric statistics remain far from the desired ranges. DE and NSGA-II can obtain only a small number of qualified samples. In contrast, the full APAM-IGLLM framework consistently generates sequences that meet the borderline conditions within a comparable number of evolutionary steps. These findings quantitatively confirm that the proposed dynamic parameter adjustment mechanism is crucial for improving convergence and effectively reducing the search complexity in the considered high-dimensional, multi-test constrained setting.

Group	Metric	Min	Max	Median	Mean	Var	Qualified samples
Group 1	Metric 1	0.0000	3.4764	1.4551	1.5069	0.0911	0
	Metric 2	0.0003	2.5039	1.0143	1.0660	0.0492	
	Metric 3	0.5819	1.1592	1.0230	1.0151	0.0016	
	Metric 4	0.0000	2.0000	0.0000	0.0060	0.0061	
	Metric 5	0.2578	1.5287	1.0156	1.0254	0.0065	
Group 2	Metric 1	0.0000	3.8781	1.3910	1.4496	0.2028	0
	Metric 2	0.0002	2.5898	1.1351	1.1266	0.0787	
	Metric 3	0.6081	1.1971	1.0523	1.0473	0.0062	
	Metric 4	0.0000	1.0000	0.0000	0.0165	0.0162	
	Metric 5	0.1728	2.0031	1.1795	1.1863	0.0368	
Group 3	Metric 1	0.0001	5.2518	1.3658	1.4717	0.3530	0
	Metric 2	0.0002	3.5020	1.2160	1.2027	0.1080	
	Metric 3	0.5841	1.1333	0.8485	0.8465	0.0022	
	Metric 4	0.0000	2.0000	0.0000	0.2929	0.2083	
	Metric 5	0.2186	1.7214	1.0412	1.0383	0.0194	
Group 4	Metric 1	0.0000	1.4101	0.2801	0.2990	0.0209	0
	Metric 2	0.0004	3.2890	1.0955	1.1199	0.0950	
	Metric 3	0.6442	1.2128	1.0428	1.0356	0.0042	
	Metric 4	0.0000	3.0000	0.0000	0.4884	0.2804	
	Metric 5	0.2137	1.6549	1.2281	1.1930	0.0267	
DE	Metric 1	0.0000	5.5768	1.4942	1.5649	0.3205	63
	Metric 2	0.1271	4.2716	1.4000	1.4770	0.2251	
	Metric 3	0.6659	1.1463	0.8980	0.9117	’0.0075	
	Metric 4	0.0000	2.0000	0.0000	0.1636	0.1395	
	Metric 5	0.2132	1.7382	1.1366	1.1194	0.0276	
NSGA-II	Metric 1	0.0001	’5.1050	1.1548	1.2353	0.3649	78
	Metric 2	0.1269	3.4141	1.1293	1.0694	0.2168	
	Metric 3	0.5887	1.2511	1.0425	1.0163	’0.0162	
	Metric 4	0.0000	2.0000	0.0000	0.3924	0.2604	
	Metric 5	’0.2749	1.5159	1.0672	1.0034	’0.0350	
APAM-IGLLM	Metric 1	0.0000	5.2568	1.1685	1.2643	0.2560	8191
	Metric 2	0.0006	4.5683	1.0280	1.1090	0.1958	
	Metric 3	0.6623	1.3225	1.2032	1.1602	0.0087	
	Metric 4	0.0000	2.0000	1.0000	0.5263	0.2518	
	Metric 5	0.2084	1.8789	1.0922	1.1109	0.0210	

### Validation of randomness testers

In this section, we evaluate the borderline samples generated by APAM-IGLLM using both classical statistical-test-based randomness test suites and the min-entropy metric. Since the fitness metrics adopted by APAM-IGLLM are themselves derived from classical statistical tests, the proposed method is primarily targeted at this class of randomness detectors. As discussed in Section “Introduction”, the NIST statistical test suite SP 800-22 has become a de facto benchmark for randomness testing, and Some other traditional test suites have been developed on top of it. Therefore, we also employ SP 800-22 as the main evaluation tool in this section. In addition to the representative tests in SP 800-22, min-entropy is used as an important metric for assessing entropy-source performance. Min-Entropy quantifies the entropy of the most probable element in a sequence, while Shannon Entropy describes the overall randomness of the sequence. Together, these two metrics characterize the randomness of a random sequence from both global and local perspectives. A random sequence consists of 8-bit binary symbols, where Shannon Entropy is expressed as

$$H(P)=\sum _i P(i) \log _a \frac{1}{P(i)}=-\sum _i P(i) \log _a P(i)$$

and Min-Entropy as

$$H_{\min }=\min _{1 \le i \le k}\left( -\log _2 p_i\right) =-\log _2 \max _{1 \le i \le k} p_i$$

**Fig. 9:**
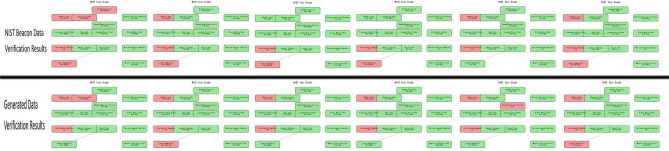
Comparison results with Nist Beacon data.

These values approaching 8 indicate that the probability distribution of each bit becomes increasingly uniform, reflecting better sequence randomness. When Min-Entropy reaches 7.85–7.95, the random sequence can pass all NIST SP 800-90B independent and identically distributed (IID) tests, with p-values far exceeding the $\alpha$=0.01 significance threshold^54^. In this experiment, 6,291 borderline samples were applied to the Min-Entropy and Shannon Entropy detectors, and the results are summarized in Table 4. The table also lists the Min-Entropy values of other commercial random number generators (RNGs), such as Red Hat’s CPU time jitter RNG (7.4528 bits per byte) and Quside’s PCIe single-quantum entropy source (6.5136 bits per byte). In addition, we extracted 50 full-entropy 0/1 random numbers (each of 4096 bits) generated by the NIST Beacon using quantum effects. We further conducted minimum entropy detection on these short sequences and 200 long sequences (each of length 102400) with strong randomness, which were derived by randomly combining the short sequences. Notably, even the Min-Entropy of these critical samples–despite their explicit statistical testing deficiencies–has already approached the NIST detection requirements closely, and in some cases, exceeds the Min-Entropy of commercial RNGs. Figure 9 illustrates the comparative performance of the generated defective data and the data obtained from NIST Beacon when evaluated against the 15 tests specified in NIST SP800-22. Each subplot, composed of 15 rectangles, represents one round of detection, where green indicates that the test passes and red indicates failure. Both datasets demonstrated strong performance, successfully passing the majority of the tests. Pre-inserted samples with borderline statistical defects can pose significant challenges to randomness testing components by enabling potentially deceptive behaviors. Such borderline-defective samples represent a more rigorous test scenario for evaluating the correctness and robustness of these components.

**Table 3:** Comparison of min-entropy results.

Source	Configuration	$\mathrm {H}_{\rm min}$	Shannon Entropy
APAM-IGLLM (this work)			
APAM-IGLLM	Windows	7.5777	7.9826
Commercial RNGs (NIST SP 800-90B IID estimates)			
IDQ quantis^55^	IID test (0 $^\circ$C)	7.8744	-
IDQ quantis^4^	Non-IID estimate^a^	7.1570	-
Microchip ECC608 NRBG^56^	Entropy source	4.0568	-
Quside PCIe One^57^	Entropy source	6.5136	-
CPU time jitter^58^	Red hat	7.4528	-
NIST Beacon(short sequences)	Quantum effects	7.4151	7.9819
NIST Beacon(long sequences)	Quantum effects	7.6438	7.9887

**Table 4:** NIST SP 800-22 test results (p-values) for six groups of NIST Beacon data. For each cell, the p-value is followed by “P” (pass) if the test satisfies the usual criterion ($p \ge 0.01$) and “F” (fail) otherwise.

Test item	Group 1	Group 2	Group 3	Group 4	Group 5	Group 6
Monobit Test	0.0108 P	0.0625 P	0.8709 P	0.9651 P	0.0151 P	0.0185 P
Cumulative Sums	0.0088 F	0.0547 P	0.6667 P	0.8831 P	0.0113 P	0.0225 P
Frequency within Block	0.0923 P	0.3207 P	0.3006 P	0.3488 P	0.6561 P	0.0398 P
Runs Test	0.8525 P	0.2679 P	0.6573 P	0.9154 P	0.5651 P	0.7967 P
Longest Run of Ones	0.3347 P	0.0965 P	0.6965 P	0.8666 P	0.6550 P	0.4342 P
Binary Matrix Rank	0.8962 P	0.9688 P	0.6532 P	0.2409 P	0.7502 P	0.9123 P
Discrete Fourier Transform	0.9087 P	0.3896 P	0.3019 P	0.3296 P	0.6057 P	0.9543 P
Non-overlapping Template	1.0000 P	1.0000 P	1.0000 P	1.0000 P	1.0000 P	1.0000 P
Overlapping Template	0.0000 F	0.0000 F	0.0000 F	0.0000 F	0.0000 F	0.0000 F
Approx. Entropy	0.0813 P	0.5872 P	0.8196 P	0.9852 P	0.4713 P	0.2093 P
Serial Test	0.0819 P	0.5885 P	0.5295 P	0.9220 P	0.4719 P	0.2130 P
Linear Complexity	0.0056 F	0.0049 F	0.0008 F	0.0026 F	0.2033 P	0.0001 F
Maurer’s Universal Test	0.9997 P	0.9990 P	0.9988 P	0.9982 P	0.9982 P	0.9997 P
Random Excursion	0.0026 F	0.1473 P	0.1684 P	0.1132 P	0.0546 P	0.1558 P
Random Excursion Variant	0.0197 P	0.0341 P	0.0623 P	0.0165 P	0.0544 P	0.0195 P

**Table 5:** NIST SP 800-22 test results (p-values) for six groups of generated data. For each cell, the p-value is followed by “P” (pass) if $p \ge 0.01$ and “F” (fail) otherwise.

Test item	Group 1	Group 2	Group 3	Group 4	Group 5	Group 6
Monobit Test	0.0138 P	0.0122 P	0.0136 P	0.0120 P	0.0148 P	0.0122 P
Cumulative Sums	0.0166 P	0.0088 F	0.0107 P	0.0135 P	0.0127 P	0.0109 P
Frequency within Block	0.0941 P	0.3189 P	0.1180 P	0.0731 P	0.1127 P	0.1621 P
Runs Test	0.0292 P	0.0311 P	0.0269 P	0.0288 P	0.0273 P	0.0771 P
Longest Run of Ones	0.4342 P	0.7024 P	0.4633 P	0.5338 P	0.6985 P	0.7441 P
Binary Matrix Rank	0.9202 P	0.9123 P	0.7871 P	0.3412 P	0.4856 P	0.2409 P
Discrete Fourier Transform	0.2397 P	0.9314 P	0.0417 P	0.9314 P	0.0253 P	0.4913 P
Non-overlapping Template	1.0000 P	1.0000 P	1.0000 P	1.0000 P	1.0000 P	1.0000 P
Overlapping Template	0.0000 F	0.0000 F	0.0000 F	0.0000 F	0.0000 F	0.0000 F
Approx. Entropy	0.0301 P	0.0363 P	0.0698 P	0.0133 P	0.0666 P	0.0341 P
Serial Test	0.0303 P	0.0365 P	0.0708 P	0.0135 P	0.0674 P	0.0344 P
Linear Complexity	0.0000 F	0.0385 P	0.0006 F	0.0400 P	0.0726 P	0.0216 P
Maurer’s Universal Test	0.9992 P	0.9992 P	0.9974 P	0.9994 P	0.9986 P	0.9996 P
Random Excursion	0.1558 P	0.0284 P	0.5151 P	0.1929 P	0.2096 P	0.3850 P
Random Excursion Variant	0.0195 P	0.1935 P	0.0357 P	0.0336 P	0.0162 P	0.0000 F

Tables 4 and 5 report the NIST SP 800-22 test results for six groups of NIST Beacon sequences and six groups of borderline samples generated by our APAM-IGLLM framework, respectively. Each cell lists the *p*-value of a specific test together with a pass/fail flag under the standard criterion ($p \ge 0.01$). Several observations can be made.

First, at the level of *which* tests are difficult, the two datasets exhibit a highly consistent pattern. For both NIST Beacon and the generated sequences, almost all core tests—including Frequency within Block, Runs Test, Longest Run of Ones, Binary Matrix Rank, Discrete Fourier Transform, Non-overlapping Template, Approximate Entropy, Serial Test and Maurer’s Universal Test—pass in all six groups. In contrast, the Overlapping Template test fails systematically for *every* group in both tables, and the Linear Complexity and Random Excursion tests are also the most challenging items, with intermittent failures appearing in both the Beacon data and our generated data. This alignment shows that the statistical “weak spots” of our borderline samples match those of a widely trusted high-quality source.

Second, in terms of *numerical* behaviour, the *p*-values of the generated sequences are of the same order of magnitude as those of NIST Beacon and are distributed over similarly broad intervals. For the majority of tests, the generated data achieve comfortably large *p*-values (often well above 0.03), rather than merely touching the threshold 0.01. Even for the more sensitive tests, such as Linear Complexity and Random Excursion, both datasets display a mixture of passes and failures with comparable *p*-value ranges. This indicates that, although APAM-IGLLM deliberately drives the sequences towards the borderline region defined by the five design metrics, the resulting samples still behave like high-quality random sequences under the full NIST SP 800-22 suite.

Taken together, these results demonstrate that our generated borderline samples not only satisfy the designed multi-metric borderline criteria, but also *closely mimic* the statistical profile of NIST Beacon when evaluated by an independent and much richer test suite. In other words, the sequences produced by APAM-IGLLM can pass (or fail) NIST SP 800-22 in almost the same way as genuine high-entropy beacon data. This makes them highly deceptive test cases for real-time randomness testers: from the perspective of standard statistical testing, they are indistinguishable from high-quality random sequences, yet they are constructed to lie near multiple critical boundaries and thus provide a much stronger stress test for embedded randomness evaluation modules.

## Discussion

The proposed APAM-IGLLM framework addresses the challenge of generating high-quality borderline test samples for randomness testers by incorporating an LLM-based controller into a genetic algorithm. The controller observes historical parameter adjustments and rewards over multiple generations and outputs new scaling factors and weight coefficients so as to maximise cumulative reward. This mechanism effectively turns the multi-metric borderline search into a dynamic game, in which the LLM implicitly learns correlations between the metrics and parameters and steers the search towards the narrow multi-constraint boundary region.

From an optimisation perspective, the experimental results confirm the benefit of this design. In the ablation studies with four static parameter configurations, at least one metric fails to converge into the desired borderline interval, and no fully qualified sample is obtained. Even when using classical metaheuristics such as Differential Evolution and NSGA-II, only a small number of qualified samples are generated. In contrast, APAM-IGLLM dynamically adapts the ten key parameters during evolution, produces a large number of borderline-qualified sequences, and exhibits more stable convergence behaviour across all five metrics. The parameter evolution curves further show that the scaling factors and weights undergo substantial adjustments in the early generations and gradually stabilise, indicating that the LLM-based controller has learnt a consistent and effective adjustment pattern. These observations demonstrate that dynamic parameter adaptation is crucial in this high-dimensional, strongly correlated, multi-test constrained setting.

To assess the general optimisation capability of APAM-IGLLM beyond the randomness-testing task, we also evaluated it on the CEC-2017 benchmark functions and compared it with several representative metaheuristic algorithms. The results show that APAM-IGLLM achieves competitive average ranks and solution quality, especially on high-dimensional and complex functions. This suggests that the proposed framework is not limited to a specific randomness-related objective, but can serve as a general high-dimensional search scheme, which supports its use as the optimisation backbone in the proposed testing scenario.

From the viewpoint of randomness assessment, the SP 800-22 and entropy evaluation results provide further insight into the nature of the generated borderline samples. Under the full NIST SP 800-22 test suite, the pass/fail patterns and *p*-value distributions of the generated samples closely resemble those of trusted NIST Beacon sequences: most core tests are consistently passed in all groups, while the same difficult items, such as Overlapping Template, Linear Complexity and Random Excursion, exhibit intermittent failures in both datasets. Moreover, the min-entropy and Shannon entropy of the generated sequences are close to those of several commercial entropy sources and approach the values obtained from NIST Beacon under SP 800-90B estimators. Taken together, these observations indicate that, although the sequences are deliberately driven towards the borderline region of multiple test criteria, they still behave like high-quality random sequences under standard statistical tests.

Despite these promising results, the present study has several limitations. First, APAM-IGLLM is specifically tailored to *statistical-test-based* randomness detectors: both the fitness function and the evaluation metrics are constructed from classical statistical tests, and our validation relies mainly on SP 800-22 and entropy-based estimators. We do not evaluate prediction-based or learning-based randomness assessors, and the effectiveness of APAM-IGLLM against such detectors remains an open question. Second, the current implementation focuses on five representative metrics and a fixed sequence length, which balances computational cost and coverage of typical failure patterns but does not exhaust all possible tests and parameter settings. Extending the framework to larger or application-specific test portfolios and to variable-length or streaming sequences is a natural direction for future work. Finally, the behaviour of the LLM-based controller depends on the underlying LLM architecture and prompt design; a more systematic study of controller robustness and lighter-weight alternatives (e.g., smaller models or specialised policy networks) will be pursued in subsequent work.

## Conclusion

To guarantee the reliability of entropy sources and the randomness of sequences, it is essential to perform randomness testing. To address the challenge of generating high-quality test data for evaluating real-time randomness testers, we proposed a novel framework, APAM-IGLLM, which combines genetic algorithms (GAs) with large language models (LLMs) to generate borderline samples that fail to satisfy one or more specified randomness criteria. The APAM-IGLLM framework transforms the problem into a multi-objective optimisation task, where the GA generates candidate borderline sequences and the LLM dynamically adjusts key parameters such as scaling factors and weight coefficients through a game-theoretic mechanism. This approach enables the generation of high-quality test data and mitigates the challenges of dimensionality and dynamic parameter adjustment in multi-constrained scenarios.

We compared the performance of the framework when integrated with different LLMs, validated the universality of the method, and analysed the decision-making characteristics of various models. The generated sequences achieve high min-entropy (7.5777 bits per byte) and Shannon entropy (7.9826 bits per byte), comparable to those of commercial entropy sources. Furthermore, we compared the performance of these sequences with random samples provided by NIST Beacon across the 15 tests specified in NIST SP 800-22; the results show highly similar pass/fail patterns and *p*-value distributions. From the viewpoint of *statistical-test-based* randomness detectors, the generated borderline samples thus effectively mimic high-quality random sequences and provide challenging, fault-detectable test cases. To support further research on randomness testing, we have begun to open-source a dataset comprising algorithmically generated borderline defect samples and random samples extracted from the NIST Beacon.

In future work, we plan to extend the APAM-IGLLM framework to support more complex and diverse randomness-testing scenarios, including non-IID sequences and multi-source entropy evaluation. Additionally, we aim to explore the integration of advanced machine learning models to further enhance the adaptability and precision of parameter tuning. Finally, we will investigate the applicability of APAM-IGLLM in other domains that require high-quality randomness testing, such as cryptographic key generation and secure communications, in order to broaden its impact and practical utility.

## Data Availability

Not all data is presented in this article; for brevity, only the most relevant results have been included. Additional data or details are available upon reasonable request by contacting the corresponding author.
